# Improving CO Oxidation Catalysis Over High Entropy Spinels by Increasing Disorder

**DOI:** 10.1002/advs.202413424

**Published:** 2025-02-20

**Authors:** Joshua D. Swindell, Gareth R.M. Tainton, Sarayute Chansai, Kerry Hazeldine, Mark A. Buckingham, Alex S. Walton, Christopher Hardacre, Sarah J. Haigh, David J. Lewis

**Affiliations:** ^1^ Department of Materials The University of Manchester Oxford Road Manchester M13 9PL UK; ^2^ Department of Chemical Engineering The University of Manchester Oxford Road Manchester M13 9PL UK; ^3^ Department of Chemistry The University of Manchester Oxford Road Manchester M13 9PL UK

**Keywords:** catalysis, CO oxidation, high entropy, molecular precursors, spinel oxides

## Abstract

Enhancing the activity and stability of earth‐abundant, heterogeneous catalysts remains a key challenge, requiring new materials design strategies to replace platinum‐group metals. Herein, it is demonstrated that increasing the configurational disorder of spinel metal oxides (M_3_O_4_, where M is a combination of Cr, Mn, Fe, Co, Ni, Cu, and Zn) leads to significant improvements in carbon monoxide (CO) oxidation performance. A substantial 63% decrease in the T_10_ value (temperature to reach 10% CO oxidation) is observed by systematically increasing the number of first‐row transition metals within the spinel oxide. Long‐term stability studies reveal that the most disordered 7‐metal spinel oxide exhibited superior resistance to catalyst deactivation compared to the 4‐metal variant, showing a decrease in activity of only 4.7% versus 12.2% during 14 h of operation. A solventless thermolysis approach is developed to synthesize a series of medium entropy spinel oxide (MESO) and high entropy spinel oxides (HESOs) from discrete, air‐stable molecular precursors. Comprehensive crystal structure determination, elemental distribution analysis, and surface characterization are conducted, establishing a clear structure‐function relationship between elemental composition, configurational disorder, and catalytic performance. This work highlights how configurational disorder can serve as an effective design principle for developing both active and stable catalysts.

## Graphical abstract

This work demonstrates that changing configurational disorder can significantly improve catalytic activity and stability. A series of high entropy spinel oxides (HESOs) are synthesized using air‐stable molecular precursors to build crystal lattices atom‐by‐atom. Spinel oxide powders are extensively characterized with a variety of advanced methods (i.e. X‐ray photoelectron spectroscopy (XPS) and atomic resolution scanning tunneling electron microscopy (STEM) imagery).

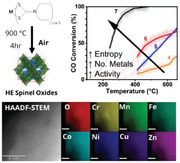

## 1 Introduction

Carbon monoxide (CO) is a highly toxic, colorless, and odorless gas produced by the incomplete combustion of fossil fuels in internal combustion engines.^[^ ^1^ ^]^ Platinum group metals (PGMs), such as Pt and Pd, show exceptional catalytic activity for CO oxidation. However, their high cost, scarcity, and susceptibility to long‐term degradation necessitate the development of more earth‐abundant, stable heterogeneous catalysts.^[^ ^2^ ^]^

**Figure 1:**
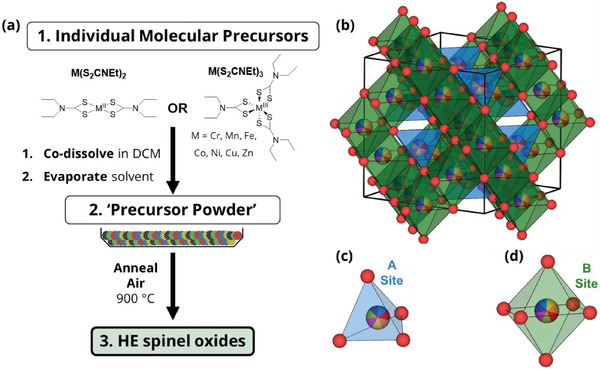
a) Schematic of the solventless thermolysis approach to form high entropy (HE) spinel oxides. b) The proposed unit cell for the 7‐metal containing HE spinel oxide (AB_2_O_4_) with assumed equimolar occupancies across the A and B sites. Red spheres = oxygen atoms and the beachball‐shaded spheres = the mixed cation sites. The lattice parameter of this unit cell is 8.33 Å as calculated vide infra. Geometry of c) tetrahedral A site, and d) octahedral B site shown.

Spinel oxides, with the general formula of AB_2_O_4_, have demonstrated promising CO oxidation activities at relatively low temperatures using earth‐abundant transition metals.^[^ ^3^ ^]^ Spinel oxides contain tetrahedral (A) and octahedral (B) coordinated cation sites (**Figure** **1**), which can both be tailored to improve catalytic performance and specificity.^[^ ^4^ ^]^ Octahedral‐terminated surfaces (e.g., (111) and (110)) are the primary contributors to catalytic activity (e.g., nitrobenzene reduction and oxygen evolution reaction) as observed simulations and through Low Energy Ion Scattering (LEIS) experiments monitoring surface cations.^[^ ^5^ ^]^ While the surface is octahedral‐terminated, subsurface cations present in tetrahedral sites are known to produce surface reconstructions and changes to catalytic selectivity and performance.^[^ ^4^, ^6^ ^]^ Catalytic activity is further influenced by the identity, valence, and distribution of metals between cation sites as well as the concentration of surface oxygen vacancies (O_v_).^[^ ^6^ ^]^ Despite promising activities and catalyst tunability, the main limitations of traditional spinel oxide catalysts remain poor long‐term stability and catalyst deactivation, particularly in the presence of water or sulfur.^[^ ^7^ ^]^

High entropy (HE) materials are a class of materials defined by high configurational entropy arising from five or more components on at least one sub‐lattice.^[^ ^8^ ^]^ HE materials offer distinct advantages over traditional materials including improved corrosion resistance, synergistic effects between active sites, and the ability to engineer lattice strain.^[^ ^8^, ^9^ ^]^ Several HE materials have been synthesized including metal alloys,^[^ ^8b,c^ ^]^ borides,^[^ ^10^ ^]^ chalcogenides,^[^ ^11^ ^]^ carbides,^[^ ^12^ ^]^ and various oxides with perovskite,^[^ ^13^ ^]^ rocksalt^[^ ^14^ ^]^ and spinel^[^ ^9^, ^15^ ^]^ structures. Since their discovery by Rost et al. in 2015,^[^ ^14d^ ^]^ HE oxides (HEO) have been applied across various fields, such as thermoelectrics,^[^ ^13b,c^ ^]^ magnetic materials,^[^ ^9^, ^14^ ^]^ and electrocatalysis.^[^ ^15c–f^ ^]^ HE oxides doped with PGMs have already been evaluated for CO oxidation, showing promising extreme thermal stability^[^ ^16^ ^]^ and use as stable single‐atom catalyst supports.^[^ ^17^ ^]^

HE spinel oxides (HESOs) are a sub‐class of HEOs that aim to combine the benefits of HE materials (stability and synergistic effects) with the unique properties of spinels (multiple cation sites and magnetic properties).^[^ ^9^, ^15^, ^18^ ^]^ For example, Yang et al. reported the synthesis of a (CrMnFeCoNi)_3_O_4_ powder using a polymeric precursor mix of citrate and metal salts annealed in air for over 18 h.^[^ ^18^ ^]^ They observed an improved specific capacity when the HESO was used as an anode for lithium‐ion batteries, compared to lower entropy systems. Recently, HESOs have exhibited improved resistance to catalytic deactivation by sulfur and water. Du et al. evaluated the SO_2_ resistance of a single HESO (ZnMgCuMnCo)Al_2_O_4_ produced via ball milling.^[^ ^19^ ^]^ The HESO was found to better resist SO_2_ poisoning in CO oxidation than conventional spinels (e.g., Co_3_O_4_) and showed good catalytic activity (210 °C produced 50% CO conversion). Zhang et al. observed the same improved resistance for the HESO (MnCuCo_3_NiFe)*_x_* O*_y_* when compared to Co_3_O_4_.^[^ ^20^ ^]^ Zhang and co‐workers then expanded this concept recently for water‐resistant spinel ACr_2_O_4_ (A = Ni, Mg, Cu, Zn, Co) catalyst for C_3_H_6_ oxidation, that outperformed single metal spinels when exposed to water during operation (stable activity for >100 h versus 20 h, respectively).^[^ ^21^ ^]^ However, to the best of our knowledge, a systematic study of PGM‐free HE oxides with increasing disorder has not yet been conducted for the CO oxidation reaction.

Conventional synthetic methods to produce HESOs include solid‐state reactions of metal salts via either ball milling, annealing, or a combination of both. Typically these processes require extreme conditions (e.g., >1000 °C for several hours) to achieve highly crystalline products but may suffer from poor stoichiometric control or phase purity, due to high material complexity.^[^ ^13^, ^14^, ^22^ ^]^ ʻBottom‐upʼ approaches via single source precursors (SSPs), which build lattices atom by atom, could offer lower thermal requirements, simplify synthetic routes, and provide access to new materials.^[^ ^23^ ^]^ SSPs are inorganic complexes with pre‐formed metal‐anion bonds (e.g., metal dithiocarbamate precursors contain metal‐sulfur bonds) that can be purified, characterized, and atomically mixed in solution. Upon heating to decomposition, volatile organics are released to leave a metal anion species (metal sulfide, selenide, or oxide). Our group has successfully used metal dithiocarbamate precursors to synthesize a range of HE materials including lanthanide oxysulfides and metal sulfides; the clear advantage of this route is that crystalline high entropy lattices can be built in an atom‐by‐atom manner, thus maximizing the disorder, hence the entropic stabilization and emergent effects afforded.^[^ ^24^ ^]^

Various SSPs have been used for the synthesis of HEO material such as mixed‐metal alkoxides^[^ ^25^ ^]^ and metal complexes with O‐donor ligands.^[^ ^26^ ^]^ However, metal oxide SSPs often face challenges, including complex SSP synthetic routes and poor stabilities.^[^ ^23b^ ^]^ Dithiocarbamate SSPs are already widely employed for metal sulfides, as they are generally air‐stable and easy to synthesize.^[^ ^23a^ ^]^ Previously, Zeng et al. demonstrated that metal dithiocarbamate precursors can also form binary and ternary metal oxides (MoO_3_ and Mo_1‐_ *_x_* W*_x_* O_3_) when decomposed in air.^[^ ^27^ ^]^ To the best of our knowledge, metal dithiocarbamate precursors have not been used to synthesize HE metal oxides.

This work explores a novel and straightforward synthetic route to HESOs via the thermolysis of metal dithiocarbamate precursor mixtures at 900 °C in air (Figure 1). The optimization of synthetic conditions by thermogravimetric analysis (TGA) to produce a series of spinel oxide (M_3_O_4_) powders containing 4, 5, 6, and 7 transition metals (Cr, Mn, Fe, Co, Ni, Cu, and Zn), characterized using X‐ray diffraction (XRD), atomic‐resolution scanning transmission electron microscopy (STEM), STEM with energy dispersive spectroscopy (STEM‐EDS) and X‐ray photoelectron spectroscopy (XPS). The catalytic performance of each spinel oxide for CO oxidation is evaluated, demonstrating the effects of the increasing disorder through the number of component elements on activity and stability.

## 2 Results and Discussion

### 2.1 Optimization of HE Spinel Oxide

Seven first‐row transition metal diethyldithiocarbamate (DDTC, [M(S_2_CNEt_2_)_n_], where M = Cr, Mn, Fe, Co, Ni, Cu, Zn, and n = 2 or 3) complexes were chosen as target precursors and synthesized by a facile synthetic route.^[^ ^24^, ^28^ ^]^ First‐row transition metals were chosen primarily due to their earth abundance and potential catalytic activity for heterogeneous catalysis. Additionally, these metals have relatively similar ionic radii and spinel oxide crystal structures, making them excellent candidates for HE spinel oxide synthesis. Each precursor was characterized by elemental analysis, Fourier transform infrared spectroscopy (FTIR), and mass spectroscopy (experimental details are found in the Supporting Infomation, chemical structures in Figure S1, Supporting Information and FTIR in Figure S2, Supporting Information).

Metal DDTC complexes have previously been thermally decomposed under argon to form metal sulfides.^[^ ^23^, ^24^, ^29^ ^]^ The thermal decomposition behavior of individual transition metal dithiocarbamates under argon, as assessed through TGA profiles, is well documented.^[^ ^24^, ^30^ ^]^ However, the effect of atmosphere (i.e., exposure to the air) or ramp rate on these complexes has been rarely studied. Singhal et al. conducted thermal decomposition studies on transition metal dithiocarbonate complexes ([M(S_2_CN(C_2_H_5_)(CH_2_CH_2_OH)] (M = Co, Ni, Cu, Zn, and Cd)) in ʻstatic airʼ and helium.^[^ ^31^ ^]^ Metal oxide products were reported for all individual metal complexes decomposed in air, except cobalt which was reduced to metallic cobalt. Therefore, this work explores the thermal decomposition behavior of transition metal DDTC complexes in both air and argon atmospheres toward the efforts to synthesize metal oxides.

**Figure 2:**
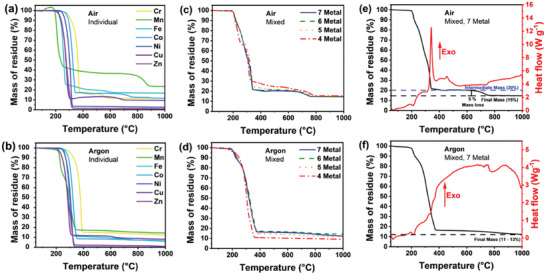
Thermogravimetric analysis (TGA) profiles for individual transition metal diethyldithiocarbamate (DDTC) precursors in air a,b) argon. c,d) refer to the pre‐ʼmixedʼ precursor powder for 4 (Cr, Mn, Fe, and Co), 5 (Cr, Mn, Fe, Co, and Ni), 6 (Cr, Mn, Fe, Co, Ni, and Cu), and 7 (Cr, Mn, Fe, Co, Ni, Cu, and Zn) metals in air and argon, respectively. Detailed TGA‐differential thermal analysis (TGA‐DSC) profiles for seven metals pre‐ʼmixedʼ in argon e) and air f) conditions highlight a high‐temperature mass loss. All TGA profiles were collected with a ramp rate of 15 °C min^−1^ between 50 and 1000 °C. For the DSC profiles shown in e,f), positive peaks represent an exothermic transition, and negative peaks an endothermic transition.

In **Figure** **2**a,b, the TGA profiles of individual DDTC precursors are overlayed for both argon and air atmospheres between 50 and 1000 °C. The TGA and differential scanning calorimetry (DSC) profiles are also shown individually in Figure S3 (Supporting Information). For molecular precursors, TGA profiles provide information on a precursor's decomposition behavior (i.e., temperature). Mass loss events correspond to the loss of gaseous decomposition products in this context. DSC provides information on reactions, phase changes, and decomposition events providing thermodynamic information from monitoring heat flow changes to a sample. Under argon, TGA profiles follow a single, clean decomposition step over a temperature range of 183 to 370 °C. In contrast, profiles in air showed multiple mass losses at high temperatures (>450 °C) after a similar initial mass loss event. Differential thermal analysis (DTA) profiles more clearly present the decomposition onset temperature and any changes in the mass loss rate over the profile. Individual DDTC precursor DTA profiles (Figure S4 and Table S1, Supporting Information) showed thermal decomposition occurred in one, two, or three stages under both atmospheres. The corresponding DSC profiles showed an endothermic transition occurred for most precursors in either atmosphere before the main decomposition step. All DSCs conducted in air showed a subsequent strong exothermic transition not observed under argon, except for Zn and Cu DDTC precursors.

During the solventless thermolysis of HE ceramics, individual precursors are co‐dissolved together in an organic solvent to facilitate atomic‐scale mixing.^[^ ^24c,d^ ^]^ Therefore, it is reasonable to assume the decomposition behavior for this pre‐ʼmixedʼ precursor powder may not be simply the average of the isolated individual components. The TGA/DSC profile of the mixed precursor powder provides insights into the decomposition behavior, which dictates the HE material formation. Figure 2c,d show the overlayed TGA profiles for pre‐mixed precursors of 4 (Cr, Mn, Fe, Co), 5 (Cr, Mn, Fe, Co, and Ni), 6 (Cr, Mn, Fe, Co, Ni, and Cu), and 7 (Cr, Mn, Fe, Co, Ni, Cu, and Zn) transition metals in both argon and air atmospheres. Figure 2e,f show the TGA/DSC behavior for the precursor powder containing seven transition metals (Figure S5, Supporting Information for other precursor powders). The TGA behavior of pre‐mixed precursor powders under argon remained similar to the individual precursors with a significant mass loss event between 200 and 260 °C. However, as highlighted by the corresponding DTA profiles in Figure S6a–c (Supporting Information), one consistent mass loss event was observed under an air atmosphere between 660 and 810 °C for the pre‐mixed precursor powders. As shown in Figure S6d (Supporting Information), this high‐temperature behavior differed from the individual precursors which showed multiple mass loss events (e.g., Co) and a wide range of mass loss event temperatures (between < 600 and 900 °C). A simple summation of the individual DTAs did not match the behavior of the pre‐mixed precursor powders. Therefore, the high‐temperature mass loss event of the pre‐mixed precursor powders can be tentatively assigned to the release of residual sulfur material preceding metal oxide formation.

To explore this further, solventless thermolysis of seven transition metals was conducted in air after the initial mass loss event at 500 °C for 1, 2, and 4 h of annealing. This was repeated under argon at 500 °C for 1 and 4 h annealing. The resulting powder X‐ray diffraction (pXRD) patterns for both conditions are shown in Figure S7 (Supporting Information). In air, at least four different crystal structures of metal sulfide and oxide mixtures were identified for 1 and 2 h of annealing. After 4 h of annealing, the pXRD pattern was not easily indexable with several additional diffraction peaks. Under argon, at least four different crystal structures were also observed at both short and long annealing times. Solventless thermolysis was then repeated above the high‐temperature mass loss event under an air atmosphere at 900 °C for 4 h. The pXRD pattern obtained was matched to the reference pattern for the crystal structure of a spinel oxide (Fe_3_O_4_, cubic, ICSD: 26410, vide infra). Alongside TGA evidence, these results suggested that other HESOs could also be synthesized at this elevated temperature. Importantly, these results show that the thermal behavior of individual precursors alone is not able to predict the behavior of the mixed powder under all reaction conditions, and there is likely to be a synergistic decomposition pathway involved.

One medium entropy spinel oxide (MESO) and three HESO (M_3_O_4_) powders were synthesized at 900 °C for 4 h via solventless thermolysis with increasing numbers of elements. The four metal (Cr, Mn, Fe, Co)_3_O_4_ should be considered a MESO as it does not meet the qualitative 5‐element requirement for HE ceramics previously defined (>0.59R in the case of a spinel oxide).^[^ ^8a^ ^]^ For simplicity, different MESO/HESO materials will be referred to as 4, 5, 6, and 7 metal or by their molecular formula as summarized in **Table** **1**. The theoretical molar configurational entropy of mixing (S_conf_) for ideal, equimolar, randomly distributed HESO compounds compare favorably with similar multi‐metallic oxides (Figure S8 and Equation S1, Supporting Information). The maximum theoretical entropy of mixing (S_conf_) for the highest entropy 7 metal HESO within this work was calculated to be S_conf_ = 6.93 J K^−1^ mol^−1^ (0.83R). This approach does not consider deviations from equally split tetrahedral and octahedral site occupancies. Some metals will occupy multiple charge states, which will drive preferential or uneven distribution between sites.^[^ ^32^ ^]^

**Table 1:** Target compositions and sample names for the spinel oxides used in this work. Bold elements represent which metals are incorporated compared to the medium entropy spinel oxide (4‐metal).

Sample	Target Composition
7 metal	(Cr Mn Fe Co **Ni Cu Zn**)_3_O_4_
6 metal	(Cr Mn Fe Co **Ni Cu**)_3_O_4_
5 metal	(Cr Mn Fe Co **Ni**)_3_O_4_
4 metal	(Cr Mn Fe Co)_3_O_4_

Site occupancies are intrinsically linked to final catalytic properties, but experimentally determining individual cation site occupancies requires extensive synchrotron‐based approaches such as X‐ray magnetic dichroism (XMCD) spectroscopy.^[^ ^32^ ^]^ The introduction of one additional element (gallium) to the 5 metal HESO (CrMnFeCoNi)_3_O_4_ was previously found to significantly alter the cation distributions of other metals such as cobalt and the magnetic properties.^[^ ^32a^ ^]^ In the absence of such direct measurements, we can theoretically consider the most likely occupancies of different metal cations using these findings for the 5 metal HESO (CrMnFeCoNi)_3_O_4_ (Supporting Information for calculation details). Cr^3+^ and Co^3+/2+^ have a strong preference for octahedral sites, whereas Ni^2+^, Cu^2+^, and Zn^2+^ have a strong preference for tetrahedral sites.^[^ ^33^ ^]^ Previous studies found that Fe^3+^ and Mn^2+/3+/4+^ could occupy both cation sites depending on the individual spinel composition. Assuming ideal cation mixing (no charge transfer, temperature, or oxygen vacancy effects), strict adherence to preferred geometries, and constant mixed Mn/Fe site occupancies, the HESO/MESO site occupancies are estimated in Table S2 (Supporting Information). Octahedral site occupancies do not change with this estimation from 4 to 7 metals, but Ni, Cu, and Zn progressively dominate tetrahedral sites. The addition of cations that prefer tetrahedral sites might drive multi‐valent, catalytically active metals (e.g., Mn and Fe) out of tetrahedral sites and into octahedral sites, which are more prevalent at the surface, thereby potentially enhancing catalytic activity.^[^ ^34^ ^]^

Additionally, the role of S_conf_ in stabilizing complex HE materials remains poorly understood and is unlikely to be the only stabilizing factor.^[^ ^35^ ^]^ Using the definition by Brahlek et al., these HESO powders can be classed as ʻhigh entropyʼ (where S_conf_ plays some role in formation) but are unlikely to be ʻentropy stabilizedʼ (where S_conf_ is critical to formation).^[^ ^36^ ^]^ Brahlek suggests that these could also be considered ʻcompositionally complex oxidesʼ but to adhere to the convention of the field the terms high or medium entropy will be used here. To determine if the final product was high entropy, two characterization metrics must be satisfied; a ʻsingleʼ crystal structure (as characterized by XRD), and a homogeneous distribution of elements across different length scales within that structure (as characterized by EDS).^[^ ^37^ ^]^

### 2.2 Crystal Structure Determination

**Figure 3:**
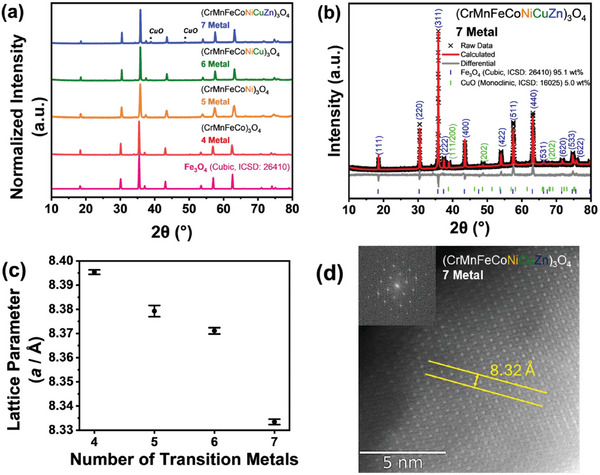
a) Powder X‐ray diffraction (pXRD) patterns for 4‐metal medium entropy spinel oxide (MESO), and high entropy spinel oxide (HESO) 5, 6, and 7‐metal powders compared to the reference spectra Fe_3_O_4_ (Cubic, ICSD: 26410). ^*^ refers to peaks associated with CuO (Monoclinic, ICSD: 67850). b) Rietveld refinement for the 7‐metal HESO powder. Error for weight percent (wt.%) is 0.3% and the weighted residual parameter (Rw) of the fit is 6.8. c) Lattice parameters, *a*, calculated from the Rietveld refinements as a function of the number of metal ions present in the cationic sub‐lattice. d) atomic resolution HAADF‐STEM image of the 7‐metal HESO along the [110] direction. The 100 lattice spacing, corresponding to lattice parameter *a*, is highlighted in the image. The inset to (d) shows the fast Fourier transform (FFT) of the image, which demonstrates the particle is being viewed along the [110] direction.

As shown in **Figure** **3**a, the pXRD patterns for the bulk powders are in good agreement with a reference inverse spinel crystal structure from the Inorganic Crystal Structure Database (ICSD) (Fe_3_O_4_, cubic, ICSD: 26410) and commercial Fe_3_O_4_ powder (Figure S9, Supporting Information) with no observable secondary crystal phases. The only exception was the observation of monoclinic CuO (ICSD: 16025) for (CrMnFeCoNiCu)_3_O_4_ and (CrMnFeCoNiCuZn)_3_O_4_ powders, which has previously been observed when Cu is doped into HESO material.^[^ ^38^ ^]^ There are no absent peaks or significant differences in the reflection intensities suggesting no preferential growth along a specific crystal axis.

To further analyze these patterns, Rietveld refinements were conducted for each sample (Figure 3; Figure S10, Supporting Information) to determine the unit cell lattice parameter (*a*/Å) and weight percent of CuO secondary phase for the 7 metal HESO. The minor CuO impurity was determined to be 5.0 ± 0.3 wt.% of the 7‐metal sample, highlighting that the spinel structure is the predominantly formed phase. For the spinel oxide contribution, the *Fd‐3m* (227) space group of a Fe_3_O_4_ parent structure with equal occupancies of all the metals at both the tetrahedral and octahedral sites was considered. Using this assumption, lattice parameters could be extracted for each MESO or HESO material, as shown in Figure 3c. The lattice parameter decreased as the number of metals increased with the lattice parameter determined for the 7 metal HESO as 8.33 Å. This could be attributed to increasing lattice distortion, or possibly oxygen and metal vacancies within the structure with increased configurational disorder.^[^ ^39^ ^]^

The crystal structure was further evaluated using atomic resolution high angle annular dark field STEM (HAADF‐STEM) imaging (Figure 3d; Figure S11, Supporting Information). The most observed zone axis was along the [110] direction, which allowed for a lattice parameter determination of 8.32 ± 0.1 Å for the 7‐metal HESO, which agreed well with the XRD above. Furthermore, this high‐resolution imaging revealed significant variation in the local orientation of the crystals (as shown in Figure S11b, Supporting Information), supporting the suggestion of no preferential crystal growth from XRD.

### 2.3 Analysis of Elemental Distributions

**Figure 4:**
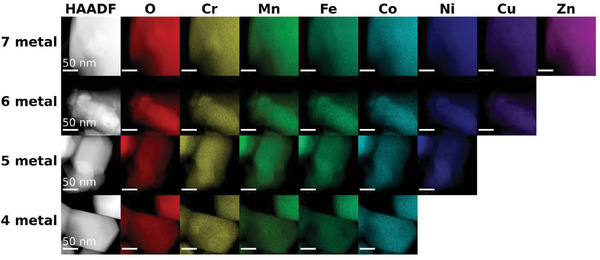
HAADF‐STEM images and energy dispersive spectroscopy (EDS) (200 kV) mapping of constituent elements within individual particles at different length scales for all 4‐ to 7‐metal‐containing spinel oxide samples. A uniform, random distribution of metals was observed with little to no segregation for all metals (except a small amount of Cr segregation) as indicated by the variation of intensities matching between different metals. The sulfur content was below 0.2 at.% or undetectable.

To investigate the elemental distribution at various length scales, both scanning electron microscopy (SEM)‐EDS (Figure S12, Supporting Information) and STEM‐EDS (**Figure** **4**; Figure S14, Supporting Information) elemental maps were collected. The EDS spectra collected using SEM (Figure S13, Supporting Information) show uniform elemental distribution at the 1 µm length scale. Importantly, for all HESO powders, no detectable sulfur was present which supports the premise that oxygen replaces sulfur in the bulk. These samples were prepared on Zn containing glass slides, which prevented accurate quantification of the metal ratios with SEM‐EDS. Furthermore, precise determination of the overall stoichiometry of the samples from either SEM‐EDS or STEM‐EDS was challenging due to the significant overlap of the transition metal L‐peaks with the oxygen K‐peak and the challenges of absorption correction for light elements. Elemental analysis at higher magnification with STEM‐EDS showed a uniform elemental distribution for all samples and co‐localization of metal cations, providing evidence for the homogenous distribution of cations across the crystal lattice. By quantifying the emission intensities from STEM‐EDS measurements (Figure S15, Supporting Information), we estimated the atomic percentages by averaging across multiple particles and the measured material compositions are summarized in Table S3 (Supporting Information).

The SEM‐EDS showed a small amount of Cu segregation for the 6 and 7 metal samples, which is consistent with the pXRD patterns showing a small amount of monoclinic CuO. Some Cr segregation was also observed by STEM‐EDS for all samples at the shortest‐length scales, but not detected at longer‐length scales (≈50 nm). One possible explanation for Cr segregation could be the higher onset temperature of decomposition for Cr(DDTC)_3_, or the incomplete dissolution of individual precursors leading to some local inhomogeneity.^[^ ^24d,e^ ^]^ Further synthetic optimization could potentially mitigate both instances of segregation, for example by changing the annealing temperature, time, or ramp rate.^[^ ^40^ ^]^

### 2.4 Surface Characterization via XPS

To better understand the surface chemistry of HESO powders pre‐catalysis, XPS was conducted on all 5 samples. XPS is surface sensitive (to depths < 10 nm), enabling probing of the chemical environment present at the surface, which is crucial for catalysis.^[^ ^41^ ^]^ Survey spectra (Figure S16, Supporting Information) identified all the expected metallic elements for each sample and were used to estimate the survey composition as compared to the bulk in Table S2 (Supporting Information). Some differences were observed between the surface and bulk, such as the increase in Mn for the 4, 5, and 6 metal spinel oxides but the results broadly agreed with the STEM‐EDS results (Figure S17, Supporting Information). High‐resolution scans of all metal core levels (Figure S18, Supporting Information) are also shown; however, spin‐orbit coupling, multiplet splitting, plasmon loss structures, and Auger peak overlaps make deconvolution of metal core levels non‐trivial, especially within HE materials.^[^ ^42^ ^]^ All core levels for the 7 metal HESO were found to have a small positive shift when calibrated to the carbon 1s (C 1s) core level. This is most likely caused by a difference in charge of the material when compared to the other samples.^[^ ^43^ ^]^

**Figure 5:**
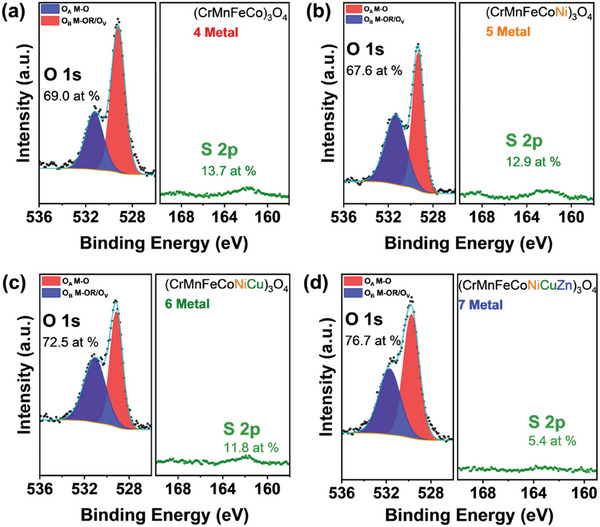
High‐resolution X‐ray photoelectron spectroscopy (XPS) scans of the O 1s and S 2p core levels for 4‐metal MESO (CrMnFeCo)_3_O_4_, and 5, 6, 7‐metal HESO (CrMnFeCoNi)_3_O_4_, (CrMnFeCoNiCu)_3_O_4_, (CrMnFeCoNiCuZn)_3_O_4_ powders a–d), respectively. The scans are plotted on the same intensity scale with no normalization to highlight the relatively low sulfur content. The fit and background are represented in blue and orange respectively. O_A_ refers to the lattice oxygen bond (M‐O) and O_B_ refers to a second oxygen environment with adventitious oxygen and M‐OR species.

High‐resolution scans of the sulfur 2p (S 2p) and oxygen 1s (O 1s) core levels were compared directly (**Figure** **5**) to assess the conversion to metal oxide. Whilst sulfur was detected on the surface (5 to 13 atom percentage (at.%)), proportionally less sulfur remained compared to surface oxygen (70 to 78 at.%). The sulfur 2p displayed two species: one at 162 eV associated with metal sulfide^[^ ^44^ ^]^ and another at 169 eV associated with sulfate^[^ ^45^ ^]^ not found in commercial Fe_3_O_4_ (Figure S19, Supporting Information). The presence of a sulfate component might indicate incomplete conversion from the sulfide to oxide conversion under the synthesis conditions used. As sulfur was not observed in detectable amounts with STEM‐EDS, this indicates the sulfur species are present primarily at the surface. Low‐degree sulfurization of spinel oxides has previously been observed to positively influence catalytic activity and cause surface reconstructions.^[^ ^46^ ^]^

The O 1s core level was fit with two oxygen environments at 529 and 531 eV attributed to lattice oxygen (O_A_, M‐O) and a second oxygen environment (O_B_, M‐OR/O_V_), respectively. Some studies refer to this higher binding energy peak as ʻdefective oxygenʼ as it encompasses several oxygen environments (e.g., adventitious oxygen) and lattice vacancies.^[^ ^47^ ^]^ The estimated composition is provided in Table S3 (Supporting Information) and general quantification tables in Table S4 (Supporting Information), demonstrating the variability of metal composition at the surface compared to the bulk. Residual sulfur, significant additional oxygen environments, and overlapping transition metal Auger peaks contribute to the observed deviations from the expected at.%.

### 2.5 Catalytic Testing

**Figure 6:**
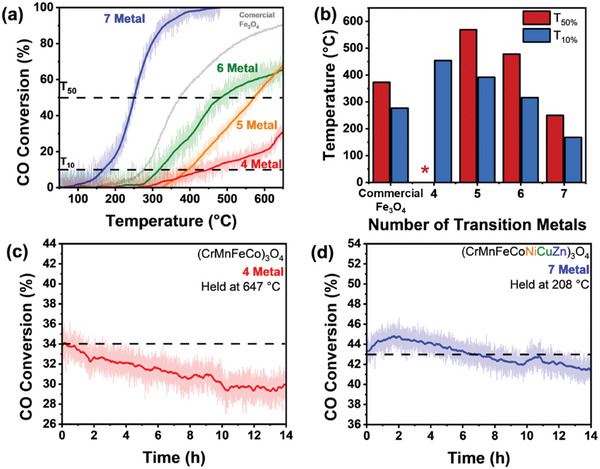
a) CO oxidation catalytic light‐off curves showing CO conversion as a function of temperature for different spinel oxide catalysts b) Comparison of T_10_ and T_50_ values as a function of the number of transition metals in each catalyst. ^*^ indicates that the 4‐metal spinel oxide did not reach 50% conversion. Catalyst stability testing for c) 4 and d) 7‐metal spinel oxides held at 647 °C and 208 °C for 14 h respectively. The dotted line indicates the initial activity at the start of the stability test, the raw data is shown behind a smoothed average. Operation conditions: total flow rate of 50 mL min^−1^, ramp rate of 10 °C min^−1^, reaction feed of 1 vol% CO/5 vol.% O_2_ and Ar balance.

The catalyst activity and stability of the spinel oxides were assessed using a plug‐flow reactor with a total flow rate of 50 mL min^−1^ under lean conditions (excess O_2_). **Figure** **6**a shows the light‐off behavior as a function of the number of transition metals calculated using Equation S2 (Supporting Information). The activity toward CO oxidation improved with an increasing number of transition metals (from 4 to 7), as shown by the required temperature for 50% and 10% conversion (T_50_ and T_10_ respectively, Figure 6b). MESO and HESOs containing 4 to 6 metals did not achieve 100% conversion, with the 4‐metal MESO found to not reach 50% conversion (hence no T_50_ can be reported). The 7‐metal HESO showed a T_10_ value of 168 °C, which was 63% lower than the 4‐metal MESO (at 454 °C), and a T_50_ value of 250 °C, which was 56% lower than the 5‐metal HESO (at 569 °C). The activity of commercially sourced Fe_3_O_4_ was also evaluated as a comparison and was found to have an intermediate T_50_ of 371 °C. These values are compared with other spinel oxides and HE oxides reported in the literature in Table S6 (Supporting Information). To adjust for catalyst mass loadings, the rate of CO conversion (µmol g^−1^ s^−1^) was calculated as a function of temperature (Figure S20 and Equation S3, Supporting Information). The same trend was observed between spinel oxides in Figure 6, with the commercially sourced Fe_3_O_4_ performing more closely to the CO conversion rates of the 5 and 6‐metal HESO. These results suggest that by changing the composition and number, the activity of a catalyst could be tuned or targeted.

To assess any potential changes post‐catalysis to residual sulfur, surface elemental composition, and oxygen vacancies, XPS spectra were collected following catalysis. Survey spectra (Figure S21, Supporting Information) were used to quantify changes to the elemental composition on the surface shown in Table S5 (Supporting Information) and graphically in Figure S22 (Supporting Information). While mostly unchanged, a notable exception was the decrease in Cr at the surface for 5‐, 6‐, and 7‐metal HESOs, paired with increases in Mn and Ni at the surface of the 6‐ and 7‐metal HESOs. These changes suggest that the cation distribution assumed in Table S2 (Supporting Information) may not hold during catalysis, where valence states are dynamic, thus further synchrotron or *operando* studies are required to fully explore these changes. Furthermore, the amount of amorphous sulfur significantly decreased for all samples to less than 2 at.%, indicating that most of the residual sulfur is likely removed either in the pre‐treatment step or during the catalysis. Upon comparing normalized high‐resolution scans of the S 2p core levels (Figure S23, Supporting Information), only one type of sulfur species assigned to sulfate remained post‐catalysis. Finally, high‐resolution scans of the O 1s core levels were collected (Figure S24, Supporting Information) to assess any changes to the second oxygen environment (O_B_, M‐OR/O_V_). Decoupling surface contamination, secondary metal‐oxygen species, and oxygen vacancies remain difficult. However, the ratio of O_B_‐to‐O_A_ decreased post‐catalysis for all samples, suggesting the perseverance of surface species or oxygen vacancies as most surface contamination is likely suppressed during the catalyst pre‐treatment. The 6‐ and 7‐metal HESOs maintained higher ratios of this second oxygen environment than the lower entropy systems. This observation suggests an increase in oxygen vacancies with higher disorder, along with a corresponding improvement of catalytic activity, as found in other HEO systems.^[^ ^20^ ^]^ Moreover, the formation of oxygen vacancies has been suggested to help regulate the generation of reactive oxygen species in catalytic oxidation reactions that proceed via the Mars–van Krevelen mechanism.^[^ ^48^ ^]^

The long‐term catalytic stability of the 4‐ and 7‐metal spinel oxide powders was also assessed. Both spinel oxides were held at a temperature lower than the T_50_ to prevent thermal runaway, for a period of 14 h (Figure 6c,d). Overall, the 4‐metal spinel oxide degraded more during the stability test compared to the 7‐metal spinel oxide (4.7% ± 0.3% versus 12.2% ± 0.4% decrease in activity, respectively). This overall change in activity was calculated by comparing the average percentage CO conversion in the first and last 0.5 h, with the relative uncertainty calculated to a 95% confidence interval. To assess the changing rate of CO conversion over time, a linear fit was applied between 0 and 2 h and 2 and 10 h (Figure S25, Supporting Information). Between 0 and 2 h, the 7‐metal spinel oxide saw an improvement in the CO conversion with an increased rate of CO conversion per hour (+ 0.62 ± 0.13% h^−1^). Conversely, the 4‐metal spinel oxide resulted in a decrease in CO conversion of –0.85 ± 0.07% h^−1^. Both spinel oxides had a decrease in the CO conversion in the 2–10 h region (−0.34 ± 0.01% h^−1^ versus–0.31 ± 0.01% h^−1^ for the 7‐ and 4‐metal spinel oxides, respectively). The 7‐metal spinel oxide also recovered some catalytic activity at ≈10 h, which could be the result of changes to surface species (e.g., sulfate/sulfide), oxygen vacancies, or metal valencies and compositional distributions under prolonged operation. In addition, the long‐term stability of the commercial Fe_3_O_4_ was assessed (Figure S26, Supporting Information) for the same period (14 h) by holding at 331 ⁰C. The decrease in catalytic activity (3.6% ± 0.3%) was comparable to the 7‐metal spinel oxide. As previously highlighted, the long‐term stability of spinel oxides is significantly impacted outside the dry conditions used in this study. Industrially realistic feeds containing catalyst poisons such as sulfur^[^ ^19^ ^]^ and water^[^ ^21^ ^]^ are known to cause significant catalyst deactivation and will be the subject of further investigations.

## 3 Conclusion

Increasing configurational disorder demonstrated significant improvements to the catalytic performance of spinel oxides for CO oxidation under dry, lean conditions (excess O_2_). The 7‐metal HESO demonstrated the best catalytic activity with a 10% CO conversion temperature (T_10_) of 168 °C, which was a 63% decrease compared to the 4‐metal MESO (at 454 °C). The 7‐metal HESO also showed better long‐term stability than the 4‐metal MESO (4.7% versus 12.2% activity decrease during 14 h of operation) indicating that incorporating more metals could help improve the catalysts’ resistance to deactivation.

A series of spinel oxides with increasing disorder was synthesized via a molecular precursor approach which required lower thermal energy input (900 °C for 4 h) than traditional solid‐state methods. Spinel oxide powders with the *Fd‐3m* (227) space group were comprehensively characterized via pXRD, atomic resolution HAADF‐STEM, SEM‐EDS and STEM‐EDS to confirm the homogenous distribution of elements across different length scales. Rietveld refinement of pXRD patterns and atomic resolution HAADF‐STEM imaging showed good agreement with the spinel structure, absence of significant secondary crystal phases, and a decreasing lattice parameter with increasing numbers of metals.

High‐resolution and survey XPS identified all composition metals at the surface with a significant lattice oxygen (M‐O) peak at 529.3 ± 0.1 eV confirming surface metal oxide. Post‐catalysis XPS suggested surface changes to the distribution of cations between geometric sites, amorphous sulfur, and oxygen vacancies could help explain differences in catalytic activity. Further optimization of the synthesis conditions could also mitigate some observed issues, such as Cr segregation at the nanoscale and CuO secondary phase formation. This work establishes a new synthetic route to HESO catalysts with tunable activity and stability through compositional and configurational disorder control. Ongoing *operando* investigations of more complex reactions (such as Fischer‐Tropsch), synchrotron‐based cation distribution analysis, and realistic reaction feeds (i.e., containing catalyst poisons such as water) will provide deeper insights into the structure‐function relationships governing these promising catalytic materials.

## 4 Experimental Section

### Chemicals

All chemicals were purchased from Sigma–Aldrich unless stated and used without further purification. Chromium(III) chloride hexahydrate (CrCl_2_·6H_2_O, ≥98%), cobalt(II) chloride hexahydrate (CoCl_2_, ≥98%, Fischer Scientific), copper(II) chloride (CuCl_2_, ≥97%), Dichloromethane (CH_2_Cl_2_, abbreviated DCM, ≥99.8%), ethanol (EtOH, ≥95.0%), hexane (C_6_H_14_, ≥97.0%), hydrochloric acid (HCl, 10% v/v), iron(III) chloride (FeCl_3_, ≥97%), iron(II, III) oxide (Fe_3_O_4_, 99.9%), nickel(II) nitrate hexahydrate (Ni(NO_3_)_2_·6H_2_O, ≥97%), manganese(II) chloride (MnCl_2_, ≥96%), methanol (MeOH, 99.9%), sodium diethyldithiocarbamate trihydrate (NaS_2_CNEt_2_·3H_2_O, abbreviated Na(S_2_CNEt_2_)_3_, ≥99.9%), zinc(II) chloride (ZnCl_2_, (CuCl_2_, ≥98%).

### Characterization

Fourier transform infrared (FTIR) spectroscopy was recorded on a Brucker alpha II Platinum ATR FTIR spectrometer. Thermogravimetric analysis (TGA) and differential scanning calorimetry (DSC) were conducted on a Mettler‐Toledo TGA/DSC1 analyzer with a ramp rate of 15 °C min−1, a temperature range of 25–1000 °C under both N_2_ and Air atmospheres. Elemental analysis (EA) was undertaken on a Thermofisher Scientific Flash 2000 Organic Elemental Analyzer. Mass spectrometry data was collected in ESI^+^ mode using an Agilent 6120 Quadrupole mass spectrometer.

X‐ray photoelectron spectroscopy (XPS) of the samples as synthesized was conducted using an ESCA 2SR high‐throughput X‐ray photoelectron spectrometer (Scienta Omicron GmbH) and the post‐catalysis was conducted using a Kratos Supra+ high‐throughput X‐ray photoelectron spectrometer (Kratos Analytical). Each system was equipped with a monochromated Al‐K_α_ (1486.6 eV) source and a hemispherical analyzer. For XPS analysis, the powders were pressed into an indium or copper foil substrate to minimize the effects of charging. The survey spectra were recorded at a pass energy of 200 eV whilst the high‐resolution core level spectra were recorded at a pass energy of 50 eV. The spectra were charge referenced to the adventitious carbon C 1s peak at 284.8 eV and analyzed using CasaXPS.^[^ ^49^ ^]^

All powder X‐ray diffraction (pXRD) patterns were collected on a Bruker D8 advance diffractometer using a Cu K_α_ radiation source (λ = 1.5419 Å).

Scanning electron microscopy‐energy dispersive X‐ray spectroscopy (SEM‐EDS) data were collected using an FEI Quanta 650 FEG SEM equipped with an Oxford Instruments X‐Max50 EDS detector operating at an acceleration voltage of 20–25 keV. SEM samples were prepared by drop casting (1–2 drops) bulk powders sonicated for 30 min in 1:1 ethanol to water solution onto a thin glass slide, coated with carbon. Scanning transmission electron microscopy (STEM) samples were also prepared in this way but deposited onto a holey carbon film coated with 200 mesh gold. Atomic resolution high angle annular dark field (HAADF) STEM images were collected using an FEI Titan “ChemiSTEM” (G2 80–200) operated at 200 kV with an aberration‐corrected probe, a 21 mrad probe convergence angle, and a HAADF inner angle of 64 mrad. EDS spectroscopy maps and the associated HAADF‐STEM images were collected using a FEI Talos F200A TEM at an operating voltage of 200 kV, equipped with a Super‐X EDS detector (4× windowless silicon drift detector). Oxford EDS Aztec software was used to analyze the SEM‐EDS data and Velox software was used for the STEM‐EDS data. Unit cell images were generated with VESTA Ver. 3.4.6.^[^ ^50^ ^]^

### Synthesis of [TM(S_2_CN(CH_2_CH_3_)_2/3_] precursors (TM = Cr, Mn, Fe, Co, Ni, Cu, Zn)

Transition metal (TM) precursors were synthesized using an adapted literature procedure.^[^ ^24^, ^28^ ^]^ Generally, sodium diethyldithiocarbamate and metal salts were separately dissolved in an appropriate solvent, with the metal salt solution slowly added to the ligand salt solution. Typically, metal complexes precipitated rapidly and were used with no further purification. The structure of each metal precursor (Figure S1, Supporting Information) was confirmed by FTIR spectroscopy (overlayed spectra shown in Figure S2, Supporting Information), elemental analysis (EA), and electrospray ionization (ESI^+^) mass spectrometry (MS). Full experimental details and characterization data can be found in the Supporting Information for each precursor.

### HE Spinel Oxide Synthesis

Following a similar procedure from previous work on metal sulfide synthesis, equimolar amounts (0.1 mmol per precursor) of transition metal diethyldithiocarbamate precursors, M(S_2_CNEt_2_)_x_ (where M = Cr, Mn, Fe, Co, Ni, Cu, Zn and x = 2 or 3), were dissolved in a minimum volume of dichloromethane.^[^ ^24^, ^51^ ^]^ The solvent was allowed to evaporate, leaving a powder of mixed precursors. This powder was transferred to a ceramic boat in a hot‐walled box furnace and heated to 900 °C (15 °C min^−1^ ramp rate) whilst exposed to static air, then held for 4 h unless stated otherwise. For inert atmosphere conditions, an argon cylinder was used to initially flush the system for 20 min at a high flow rate (>250 cm^3^ min^−1^) which was reduced to between 60 and 150 cm^3^ min^−1^ when annealing. The sample was allowed to cool to room temperature before removal. All powders were subsequently ground by hand for further characterization analysis. The target compositions of the spinel oxides synthesized in this work are summarized in Table 1 below.

### Catalytic Testing

CO oxidation catalytic testing was conducted in a quartz tubular reactor (4 mm inner diameter, 6 mm outer diameter). Typically, ≈35 mg of spinel oxide powder catalyst was packed and held in place between plugs of quartz wool, and a K‐type thermocouple was placed in the center of the catalyst bed. The reactor was then placed inside a Carbolite tube furnace. The catalysts were pretreated in 20 vol.% O_2_/Ar at a flow rate of 50 mL min^−1^ at 450 °C for 1 h at a ramp rate of 10 °C min^−1^ before the reaction to reduce surface contamination as seen previously.^[^ ^3c^ ^]^ The feed gas consisted of 1 vol.% CO (BOC, 99.98%), 5 vol.% O_2_ (BOC, 99.98%), and Ar (BOC, 99.98%) balance was introduced to the reactor heated from 50 to 650 °C at a ramp rate of 10 °C min^−1^. Each of the gases in the feed mixture was controlled individually by mass flow controllers and the total gas flow rate was 50 cm^3^ min^−1^. For the long‐term stability measurements, the same feed conditions were used and the temperature held (647, 331, and 208 ⁰C for the 4‐metal MESO, Fe_3_O_4_, and 7‐metal HESO respectively). The reactants and products were monitored by an HPR20 QIC Hiden Analytical mass spectrometer with an electron impact ionization and with ion currents set for mass‐to‐charge ratio (m/*Z*) as for 18 (H_2_O), 28 (CO/CO_2_), 32 (O_2_), 36 (Ar), and 44 (CO_2_). CO conversion calculations were discussed in the Supporting Information following Equations S2 and S3 (Supporting Information).

### Statistical Analysis

The data presented were the raw data unless otherwise stated below or in the text. Normalization was typically conducted between 0 and 1 as shown in pXRD patterns within Figure 3a, Figures S7 and S9 (Supporting Information), FTIR data in Figure S2 (Supporting Information), and XPS data in Figure S23 (Supporting Information). Averaging and statistical analysis of estimated compositions was stated in the supporting information or within the text where appropriate. All calculated errors were ensured to be within a 95% confidence interval.

## Conflict of Interest

The authors declare no conflict of interest.

## Supporting information



Supporting Information

## Data Availability

The data that support the findings of this study are available from the corresponding author upon reasonable request.
