# Supplementary material for: Improving CO Oxidation Catalysis Over High Entropy Spinels by Increasing Disorder
Source: Adv Sci (Weinh). 2025 Feb 20;12(15):2413424. doi: 10.1002/advs.202413424 (PMC12005821; doi:10.1002/advs.202413424)
Supplement: Supplementary file 1 — Supporting Information [file ADVS-12-2413424-s001.docx]

Supporting Information

Improving CO Oxidation Catalysis Over High Entropy Spinels by Increasing Disorder

Joshua D. Swindell, Gareth R.M. Tainton, Sarayute Chansai, Kerry Hazeldine, Mark A. Buckingham, Alex S. Walton, Christopher Hardacre, Sarah J. Haigh, and David J. Lewis*

**Synthesis of Transition Metal (TM) diethyldithiocarbamate Precursors**

**Synthesis of Tris(N,N-diethyldithiocarbamato) Chromium(III) ([Cr(S_2_CNEt_2_)_3_])**

[Na(S_2_CNEt_2_)] (12.2 g, 54 mmol) was dissolved in 100 mL H_2_O. Separately, CrCl_2_.6H_2_O (4 g, 18 mmol) was dissolved in 100 mL H_2_O and 2M HCl was added dropwise until the solution was ~ pH 4 under constant stirring. The metal chloride solution was slowly added to the ligand solution at room temperature. The reaction mixture was left under constant stirring for 1 h until a light blue precipitate was formed. The crude product was isolated by vacuum filtration and thoroughly washed with H_2_O, methanol, and hexane then left to dry *in vacuo* for 2 h. The dry crude product was further purified by column chromatography on silica-eluting dichloromethane, collecting the rapidly eluting dark blue band. The solvent was removed under vacuum to yield a dark blue solid. Yield 10 %. EA measured % (expected % for Cr(C_15_H_30_N_3_S_6_)); C, 36.3 (36.5); H, 6.1 (6.0); N, 8.3 (8.5); S, 38.7 (38.6). FTIR (cm^-1^): 489, 573, 595, 734, 781, 845, 912, 991, 1072, 1130, 1144, 1208, 1266, 1295, 1352, 1372, 1430, 1448, 1485, 2868, 2927, 2979. ESI^+^ MS *m/z* (M^+^Na^+^): 519.00.

**Synthesis of Tris(N,N-diethyldithiocarbamato) Manganese(III) ([Mn(S_2_CNEt_2_)_3_])**

A solution of MnCl_2_ (1.27 g, 10 mmol) in 50 mL MeOH was slowly added to a solution of [Na(S_2_CNEt_2_)] (6.76 g, 30 mmol) in 150 mL MeOH at room temperature under constant stirring. The reaction mixture was left for 2 h before a black precipitate was formed. The crude product was isolated by vacuum filtration and thoroughly washed with H_2_O, methanol, and hexane. The crude product was purified by dissolving in 20 mL DCM with stirring for a short period, followed by the rapid addition of 10 mL EtOH. Stirring was continued for a further 5 min, and the solution was left to stand for 10 min. The pure product was vacuum filtrated, thoroughly washed as before, and then left to dry *in vacuo* at room temperature overnight. Yield 63 %. EA measured % (expected % for Mn(C_15_H_30_N_3_S_6_) . 0.5H_2_O); C, 35.3 (35.4); H, 6.4 (5.9); N, 8.1 (8.3); S, 37.1 (37.7). FTIR (cm^-1^): 571, 596, 782, 844, 912, 988, 1069, 1094, 1143, 1205, 1266, 1295, 1348, 1377, 1424, 1440, 1457, 1487, 1509, 2865, 2926, 2971. ESI^+^ MS *m/z* (M-H^+^): 499.0.

**Synthesis of Tris(N,N-diethyldithiocarbamato) Iron(III) ([Fe(S_2_CNEt_2_)_3_])**

A solution of FeCl_3_ (1.62 g, 10 mmol) in 50 mL MeOH was slowly added to a solution of [Na(S_2_CNEt_2_)] (6.76 g, 30 mmol) in 50 mL MeOH at room temperature under constant stirring. The reaction mixture was left for 1 h before a black precipitate was formed. The crude product was isolated by vacuum filtration and thoroughly washed with H_2_O, methanol, and hexane. The product was vacuum filtrated and then left to dry *in vacuo* at room temperature overnight. Yield 54 %. EA measured % (expected % for Fe(C_15_H_30_N_3_S_6_)); C, 33.5 (33.4); H, 5.6 (5.6); N, 7.7 (7.8); S, 35.7 (35.6). FTIR (cm^-1^): 498, 571, 606, 777, 847, 912, 995, 1071, 1095, 1146, 1206, 1271, 1299, 1352, 1377, 1434, 1452, 1501, 1566, 2868, 2927, 2980. ESI^+^ MS *m/z* (M+H^+^): 500.01.

**Synthesis of Tris(N,N-diethyldithiocarbamato) Cobalt(III) ([Co(S_2_CNEt_2_)_3_])**

A solution of CoCl_2_.6H_2_O (2.30 g, 10 mmol) in 50 mL MeOH was slowly added to a solution of [Na(S_2_CNEt_2_)] (6.76 g, 30 mmol) in 150 mL MeOH at room temperature under constant stirring. The reaction mixture was left for 2 h before a green precipitate was formed. The crude product was isolated by vacuum filtration and thoroughly washed with H_2_O, methanol, and hexane. The crude product was purified by dissolving in 20 mL DCM with stirring for a short period, followed by the rapid addition of 10 mL EtOH. Stirring was continued for a further 5 min and the solution was left to stand for 10 mins. The pure product was vacuum filtrated, thoroughly washed as before, and then left to dry *in vacuo* at room temperature overnight. Yield 63 %. EA measured % (expected % for Co(C_15_H_30_N_3_S_6_) . 0.5H_2_O ); C, 34.8 (35.1); H, 5.9 (6.0); N, 8.1 (8.2); S, 37.2 (37.4). FTIR (cm^-1^): 564, 581, 602, 784, 846, 913, 996, 1059, 1074, 1132, 1147, 1211, 1263, 1292, 1351, 1373, 1431, 1449, 1482, 2865, 2926, 2971. ESI^+^ MS *m/z* (M+Na^+^): 526.00.

**Synthesis of Bis(N,N-diethyldithiocarbamato) Nickel(II) ([Ni(S_2_CNEt_2_)_2_])**

A solution of Ni(NO_3_)_2_.6H_2_O (3.23 g, 10 mmol) in 50 mL MeOH was slowly added to a solution of [Na(S_2_CNEt_2_)] (5 g, 22 mmol) in 50 mL MeOH at room temperature under constant stirring. The reaction mixture was left for 1 h before a green precipitate was formed. The product was isolated by vacuum filtration and thoroughly washed with H_2_O, methanol, and hexane. This was left to dry *in vacuo* at room temperature overnight. Yield 94 %. EA measured % (expected % for Ni(C_10_H_20_N_2_S_4_)); C, 33.9 (33.8); H, 5.8 (5.7); N, 7.8 (7.9); S, 36.2 (36.1). FTIR (cm^-1^): 420, 492, 573, 602, 781, 851, 912, 991, 1077, 1093, 1155, 1206, 1274, 1299, 1352, 1377, 1431, 1517, 1564, 2870, 2929, 2972. ESI^+^ MS *m/z* (M+Na^+^): 376.98.

**Synthesis of Bis(N,N-diethyldithiocarbamato) Copper(II) ([Cu(S_2_CNEt_2_)_2_])**

A solution of CuCl_2_ (1.50 g, 11 mmol) in 50 mL MeOH was slowly added to a solution of [Na(S_2_CNEt_2_)] (5 g, 22 mmol) in 50 mL MeOH at room temperature under constant stirring. The reaction mixture was left for 1 h before a brown precipitate was formed. The product was isolated by vacuum filtration and thoroughly washed with H_2_O, methanol, and hexane. This was left to dry *in vacuo* at room temperature overnight. Yield 52 %. EA measured % (expected % for Cu(C_10_H_20_N_2_S_4_)); C, 33.5 (33.4); H, 5.6 (5.6); N, 7.7 (7.8); S, 35.7 (35.6). FTIR (cm^-1^): 498, 571, 606, 777, 847, 912, 995, 1071, 1095, 1146, 1206, 1271, 1299, 1352, 1377, 1434, 1452, 1501, 1566, 2868, 2927, 2980. ESI^+^ MS *m/z*: 358.99 (M-H^+^).

**Synthesis of Bis(N,N-diethyldithiocarbamato) Zinc(II) ([Zn(S_2_CNEt_2_)_2_])**

A solution of ZnCl_2_ (1.54 g, 11 mmol) in 50 mL MeOH was slowly added to a solution of [Na(S_2_CNEt_2_)] (5 g, 22 mmol) in 50 mL MeOH at room temperature under constant stirring. The reaction mixture was left for 1 h before a white precipitate was formed. The product was isolated by vacuum filtration and thoroughly washed with H_2_O, methanol, and hexane. This was left to dry *in vacuo* at room temperature overnight. Yield 81 %. EA measured % (expected % for Zn(C_10_H_20_N_2_S_4_)); C, 33.4 (33.2); H, 5.5 (5.6); N, 7.7 (7.8); S, 35.4 (35.4). FTIR (cm^-1^): 426, 506, 563, 612, 777, 840, 912, 991, 1071, 1095, 1144, 1201, 1271, 1297, 1354, 1377, 1428, 1450, 1499, 2870, 2929, 2966. ESI^+^ MS *m/z*: 360.99 (M-H^+^).

**A note on cobalt and manganese diethyldithiocarbamate complexes**

For the cobalt and manganese diethyldithiocarbamate complexes, recent work by our group has experimentally confirmed that the [M^2+^(S_2_CNEt_2_)_2_] readily oxidizes to [M^3+^(S_2_CNEt_2_)_3_] or forms an M^2+^_2_O_2_(S_2_CNEt_2_)_2_ bridging dimer.^[1]^ This occurred even with 2 equivalents of diethyldithiocarbamate ligands, so [M^3+^(S_2_CNEt_2_)_3_] complexes were intentionally synthesized here.

**Calculation of Molar Configurational Entropy (S_conf_)**

As established in previous work, to calculate molar S_conf_, the metal elements are assumed to be randomly distributed across the metal sub-lattice.^[2]^ The fraction that this sub-lattice occupies in the total unit cell must be accounted for and is often a source of error when calculating S_conf_. The following equation should be used:

$S_{conf}= -R\frac{N^{M}}{N}\sum\frac{N^{X}}{N^{M}}\ln\frac{N^{X}}{N^{M}}$ (S1)

Where N^X^ is the number of metal atoms X, N^M^ is the total number of atoms in the metal sub-lattice, N is the number of atoms in total in the unit formula and R is the ideal gas constant.

**Calculation of estimated site occupancies**

To estimate the cation site occupancies in a spinel structure of AB_2_O_4_ where tetrahedral (A) and octahedral (B) sites require synchrotron-based approaches, some assumptions were applied:

1. Ideal equimolar cation mixing and complete miscibility with no phase segregation
2. No temperature effects
3. No charge transfer events
4. Stoichiometric oxygen occupancy considering no site vacancies
5. Cr^3+^ and Co^3+/2+^ have a strong preference for octahedral sites such that we will assume exclusive octahedral occupancy.
6. Ni^2+^, Cu^2+^, and Zn^2+^ have a strong preference for tetrahedral sites such that we will assume exclusive tetrahedral occupancy.
7. Fe^3+^ and Mn^2+/3+/4+^ have mixed site occupancy using the values obtained from Johnstone *et al.* for the 0% Ga sample.^[3]^

There are 3 cations per formula unit, and each metal contributes 3/n cations per formula unit where n is the number of cations added.

e.g. (CrMnFeCo)_3_O_4_ has 0.75 cations per formula unit (f.u.) that add to maintain 3 cations overall.

Considering the assumptions above, the distribution of cations is based on:

Cr = 100% B

Co = 100% B

Ni = 100% A

Cu = 100% A

Zn = 100% A

Mn = 10% A, 90% B

Fe = 54% A, 46% B

Multiplying the cations/ f.u. by the site preference fraction above to gain an initial distribution.

e.g. 4-Metal MESO for Mn:

$$A site occupancy= 0.75\times0.1=0.075$$

To maintain ideal stoichiometry, the values were then normalized for a total A site occupancy of 1 and B site occupancy of 2.

e.g. 4-Metal MESO:

$$Initial A site occupancy of \mathbf{Mn}= 0.075$$

$$Initial A site occupancy of \mathbf{Fe}= 0.405$$

$$Total site occupancy initially calculated= 0.48 \neq1$$

$$Normalized A site occupancy for \mathbf{Mn}= \frac{1}{0.48} \times0.075=\mathbf{0.16}$$

The final, estimated, and normalized occupancies/compositions are reported in **Table S2**. These calculations are intended to be used as an approximation and will vary outside the assumptions made here.

**Catalysis calculations**

To calculate the CO conversion (%) the following equation was used:

$X_{CO}\left( \% \right)= \frac{(C_{CO,in}-C_{CO, out})}{C_{CO, in}} \times100 \%$ (S2)

Where *C_CO,in_* and *C_CO,out_* represent the inlet and outlet concentrations of carbon monoxide (m/z = 28), respectively. A correction factor was applied for fragmented CO_2_ contributing approximately 9.5% to the m/z = 28 signal. The carbon monoxide concentrations were baseline corrected and then normalized to the Kr (m/z = 84) signal.

Due to a slight variation between mass loadings, the rate of CO conversion was calculated to decouple the effect of catalyst loading using the following equation:

$Rate \left( mol g^{-1} s^{-1} \right)= \frac{{CO}_{consumed}\left( ppm \right)}{mass \left( g \right)} \times\frac{TFR \left( {cm}^{3} s^{-1} \right)}{24465 ({cm}^{3} {mol}^{-1})} \times{10}^{-6} ({ppm}^{-1})$ (S3)

Where the rate is the change of CO conversion, CO_consumed_ is the amount of CO consumed relative to the input CO (10000 ppm), mass is the catalyst loading, and the TFR is the total flow rate. The constant 24465 results from the ideal gas law at room temperature (25 ⁰C) and normal pressure (1 atm).

The mass loadings for the standard CO oxidation experiment were:

| **Sample** | **Mass used/ mg** |
| --- | --- |
| 7 metal | 42 |
| 6 metal | 29 |
| 5 metal | 38 |
| 4 metal | 34 |
| Fe_3_O_4_ | 51 |

**Supplementary Figures**

**
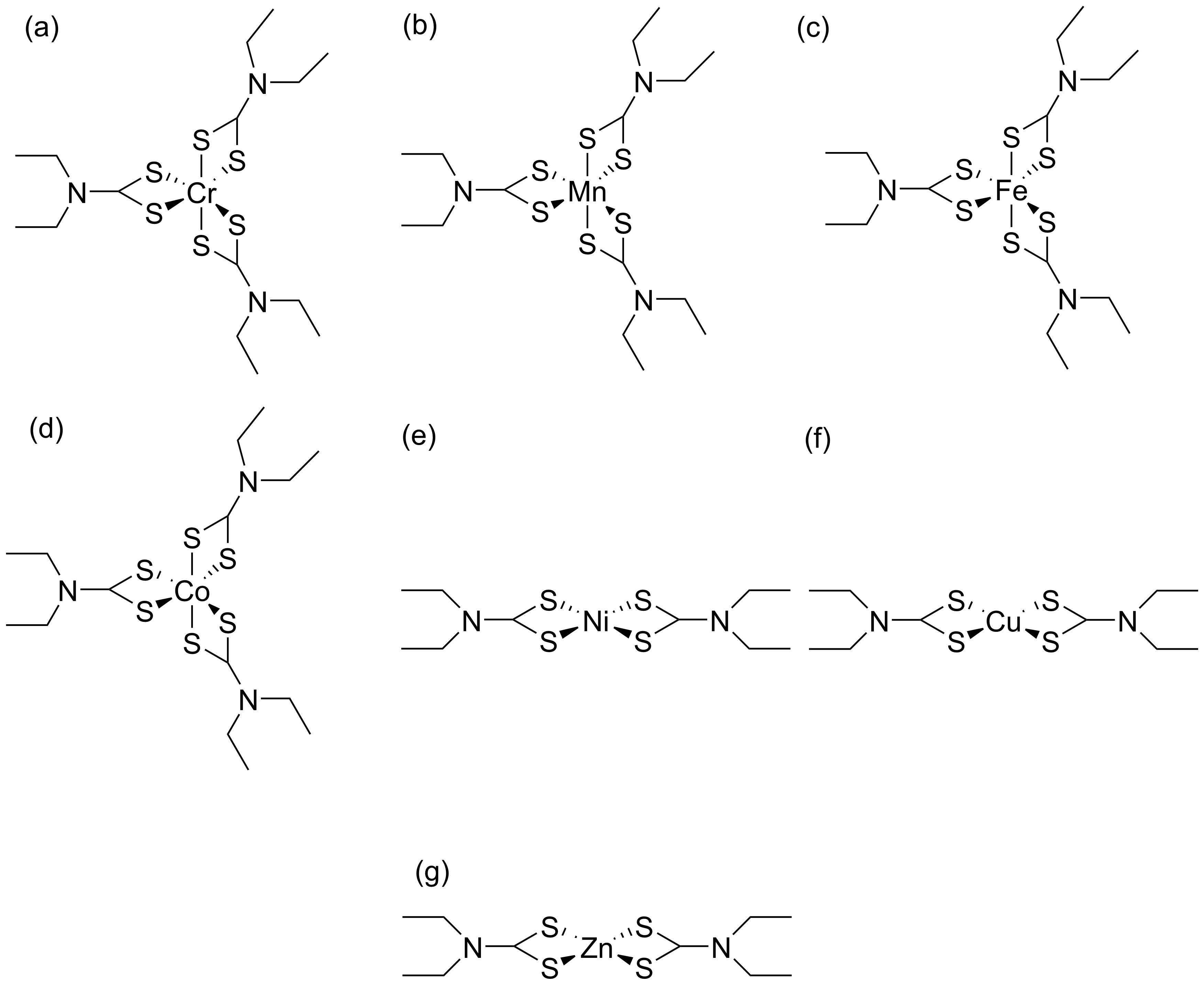
**

**Figure S1.** Chemical structures of all 9 metal diethyldithiocarbamate precursors synthesized with the formula [M(S_2_CNEt_2_)n] where n = 2 or 3 and M = Cr, Mn, Fe, Co, Ni, Cu or Zn shown (a) to (g) respectively.

**
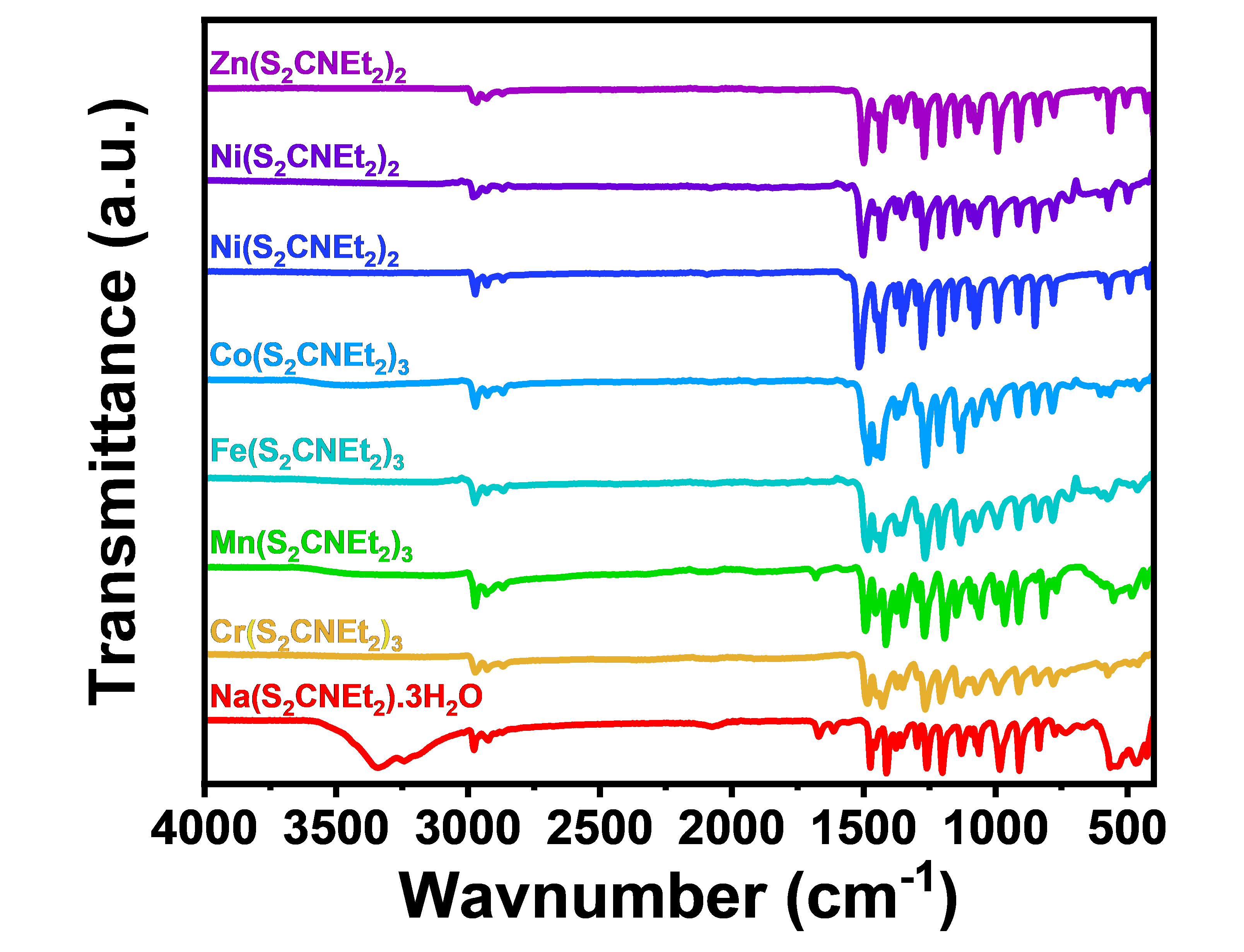
**

**Figure S2.** FTIR of all 9 precursors compared to the ligand (Na(S_2_CNEt_2_).3H_2_O).

**
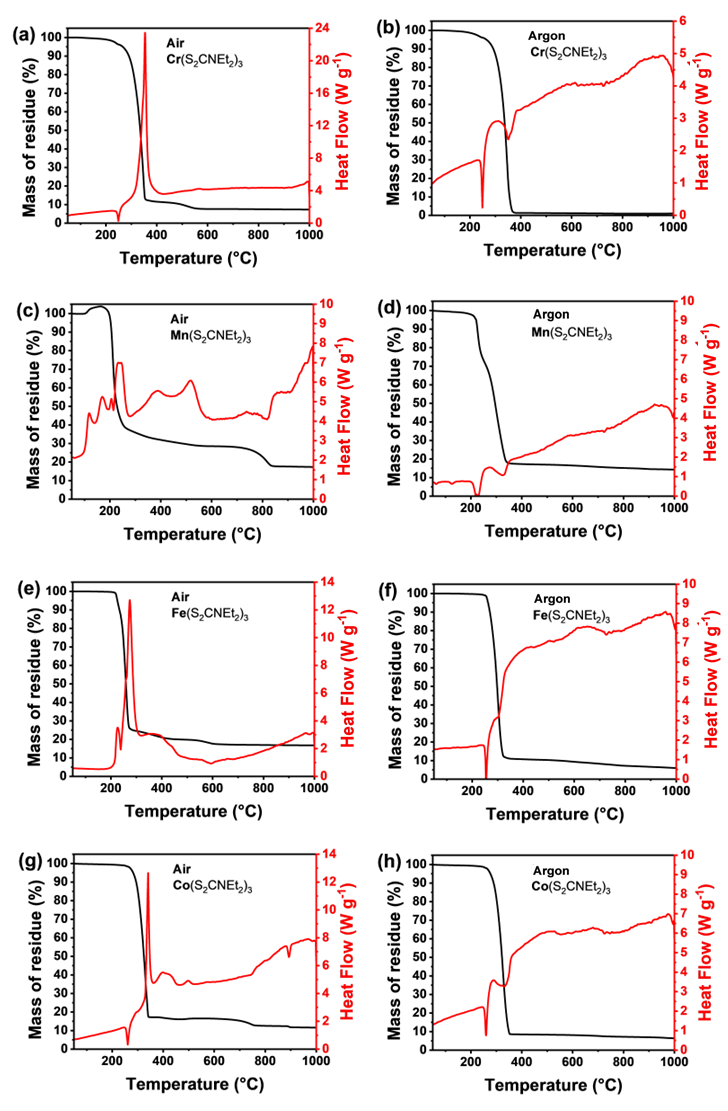
**

**
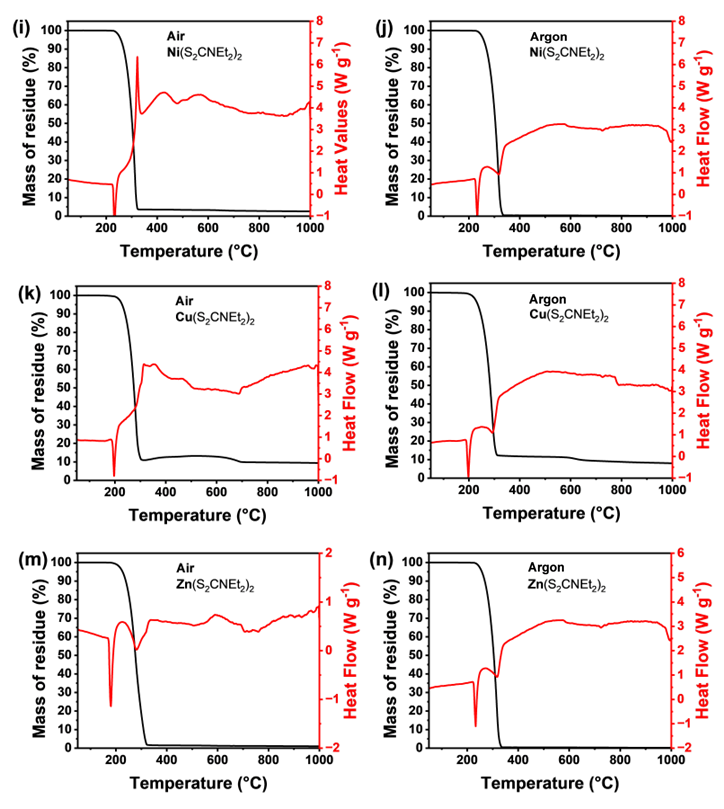
**

**Figure S3.** TGA/DSC profiles of each transition metal DDTC complex under inert and air atmospheres. For the DSC profiles, positive peaks represent an exothermic transition, and negative peaks an endothermic transition.

**
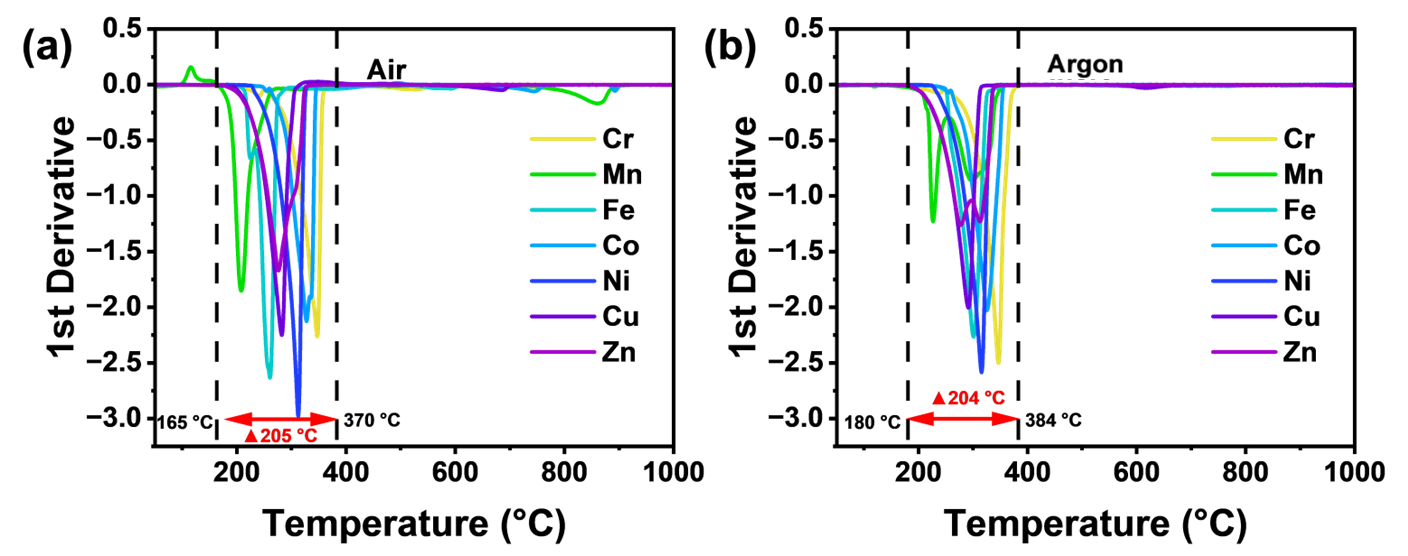
**

**Figure S4.** Overlayed differential thermal analysis (DTA) profile of each precursor for (a) air and (b) inert conditions. The first derivative of the respective TGA profile is plotted against the temperature applied.

**Table S1.** The onset and final temperatures of the first mass loss event for different individual precursors from DTA plots in Figure S4.

| **Sample** | **Air** | | **Inert** | |
| --- | --- | --- | --- | --- |
|  | **Onset / °C** | **Final / °C** | **Onset / °C** | **Final / °C** |
| Cr(DDTC)­_3_  Mn(DDTC)­_3_  Fe(DDTC)­_3_  Co(DDTC)­_3_  Ni(DDTC)­_2_  Cu(DDTC)­_2_  Zn(DDTC)­_2_ | 252  165  212  251  226  192  178 | 370  178  295  351  332  322  337 | 249  180  232  241  224  184  183 | 384  364  361  363  346  323  355 |


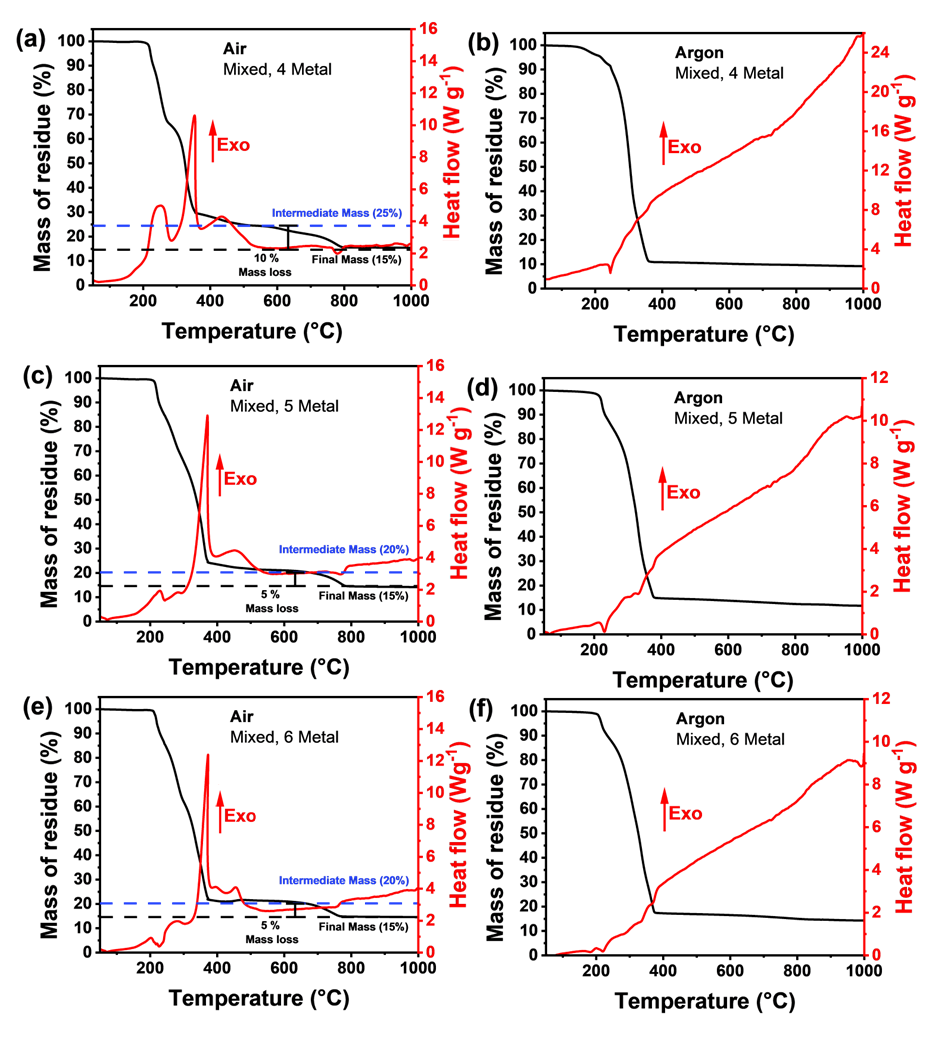


**Figure S5.** Individual TGA/DSC profiles of 4, 5, and 6 metal ‘precursor powders’ in air and inert conditions.


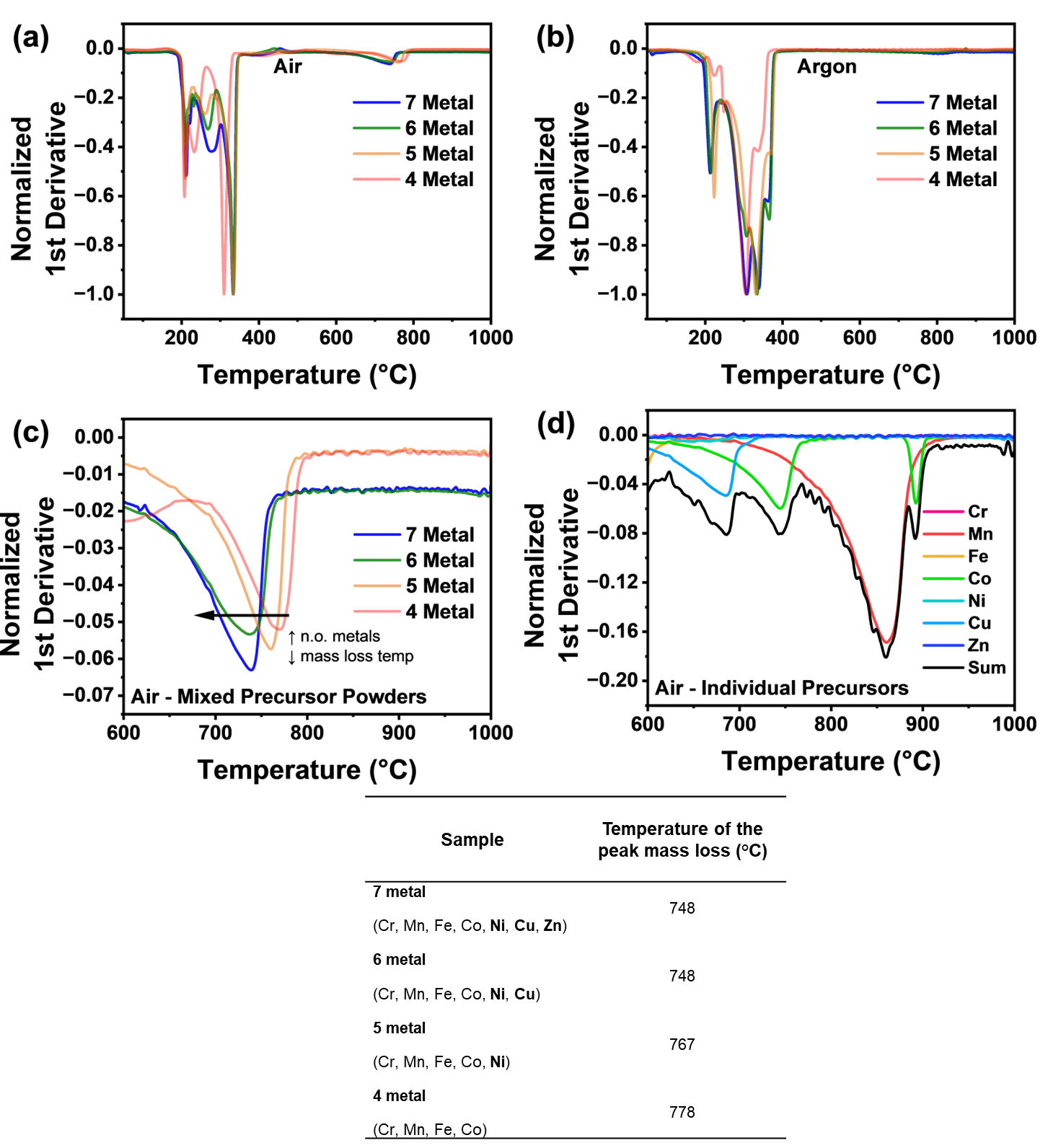


**Figure S6.** Overlayed DTA profiles of 4, 5, 6, and 7 metal-mixed ‘precursor powders’ in air (a) and inert (b) conditions. (c) shows an inset of the high-temperature region for each mixed precursor powder and compared with a summation of all 7 individual precursor DTA profiles in air within the same region. The temperature of the peak mass loss for each mixed precursor powder is presented with decreasing dependence on the increasing number of metals.


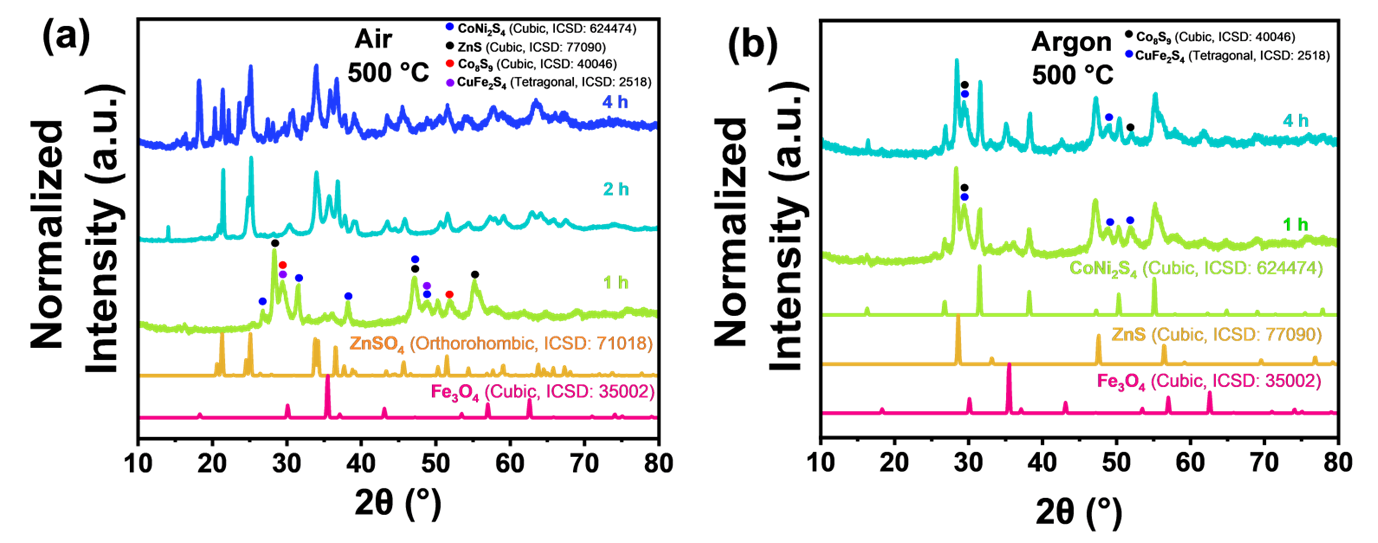


**Figure S7.** pXRD of 7 metal precursor powders (Cr, Mn, Fe, Co, Ni, Cu, Zn) annealed at 500 °C for 1, 2, or 4 hours in (a) air and (b) argon atmospheres. Dots represent other alternative phases for major peaks not assigned within the reference patterns below the obtained data.

**
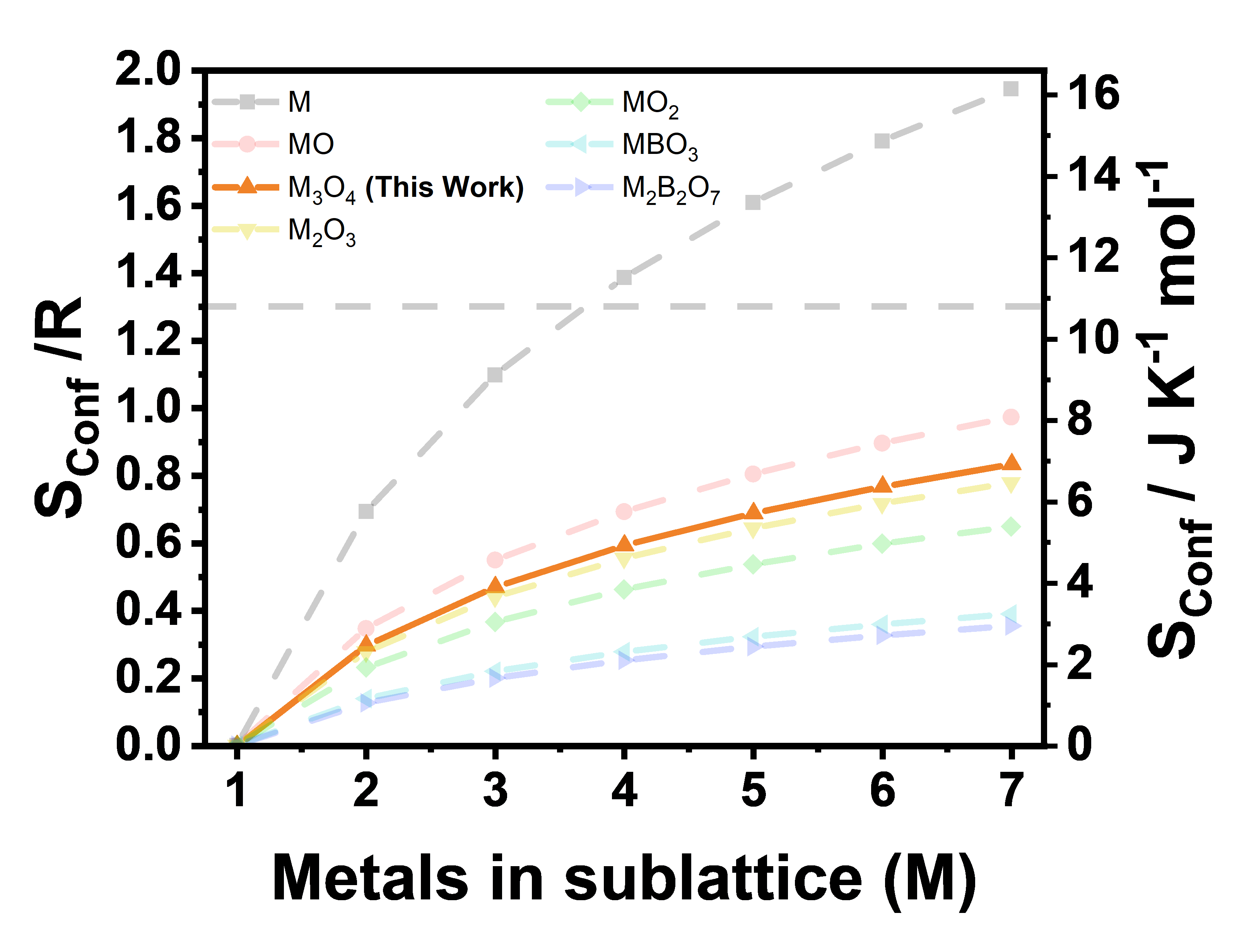
**

**Figure S8.** Comparison of calculated configurational entropy as a function of the number of doped metals for various metal oxides and alloys. The line represents the commonly used merit of high entropy for alloys of 1.5R. This is less relevant to high entropy ceramics which have multiple sublattices and can form stable high entropy materials below this metric.

### **Table S2.** (a) Estimated cation site occupancies calculated from the previous section titled ‘Calculation of estimated site occupancies’. **A** **site** refers to tetrahedrally coordinated sites and **B site** refers to octahedrally coordinated sites following the stoichiometric formula AB_2_O_4_. (b) The estimated cation composition from these calculated values.

| **Sample** | **A site** | | | | | **B site** | | | |
| --- | --- | --- | --- | --- | --- | --- | --- | --- | --- |
|  | **Mn** | **Fe** | **Ni** | **Cu** | **Zn** | **Cr** | **Mn** | **Fe** | **Co** |
| **7 metal**  (Cr, Mn, Fe, Co, **Ni**, **Cu**, **Zn**) | 0.03 | 0.15 | 0.27 | 0.27 | 0.27 | 0.60 | 0.54 | 0.27 | 0.60 |
| **6 metal**  (Cr, Mn, Fe, Co, **Ni**, **Cu**) | 0.04 | 0.21 | 0.38 | 0.38 | - | 0.60 | 0.54 | 0.27 | 0.60 |
| **5 metal**  (Cr, Mn, Fe, Co, **Ni**) | 0.06 | 0.33 | 0.61 | - | - | 0.60 | 0.54 | 0.27 | 0.60 |
| **4 metal**  (Cr, Mn, Fe, Co) | 0.16 | 0.84 | - | - | - | 0.60 | 0.54 | 0.27 | 0.60 |


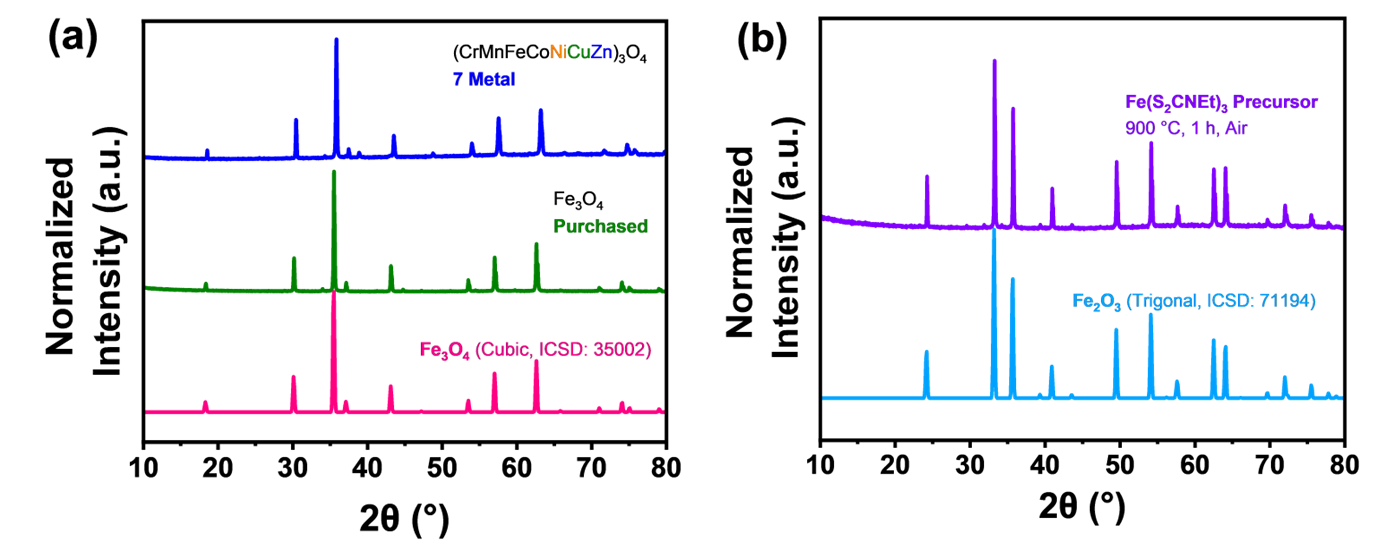


**Figure S9.** (a) pXRD of precursor powder of 7 metals (Cr, Mn, Fe, Co, Ni, Cu, Zn) annealed at 900 °C for 4 hours compared to commercially sourced Fe_3_O_4_. (b) pXRD of the Fe(DDTC)_3_ precursor annealed in air for 1 hour compared to the Fe_2_O_3_ (Trigonal, ICSD: 71194).


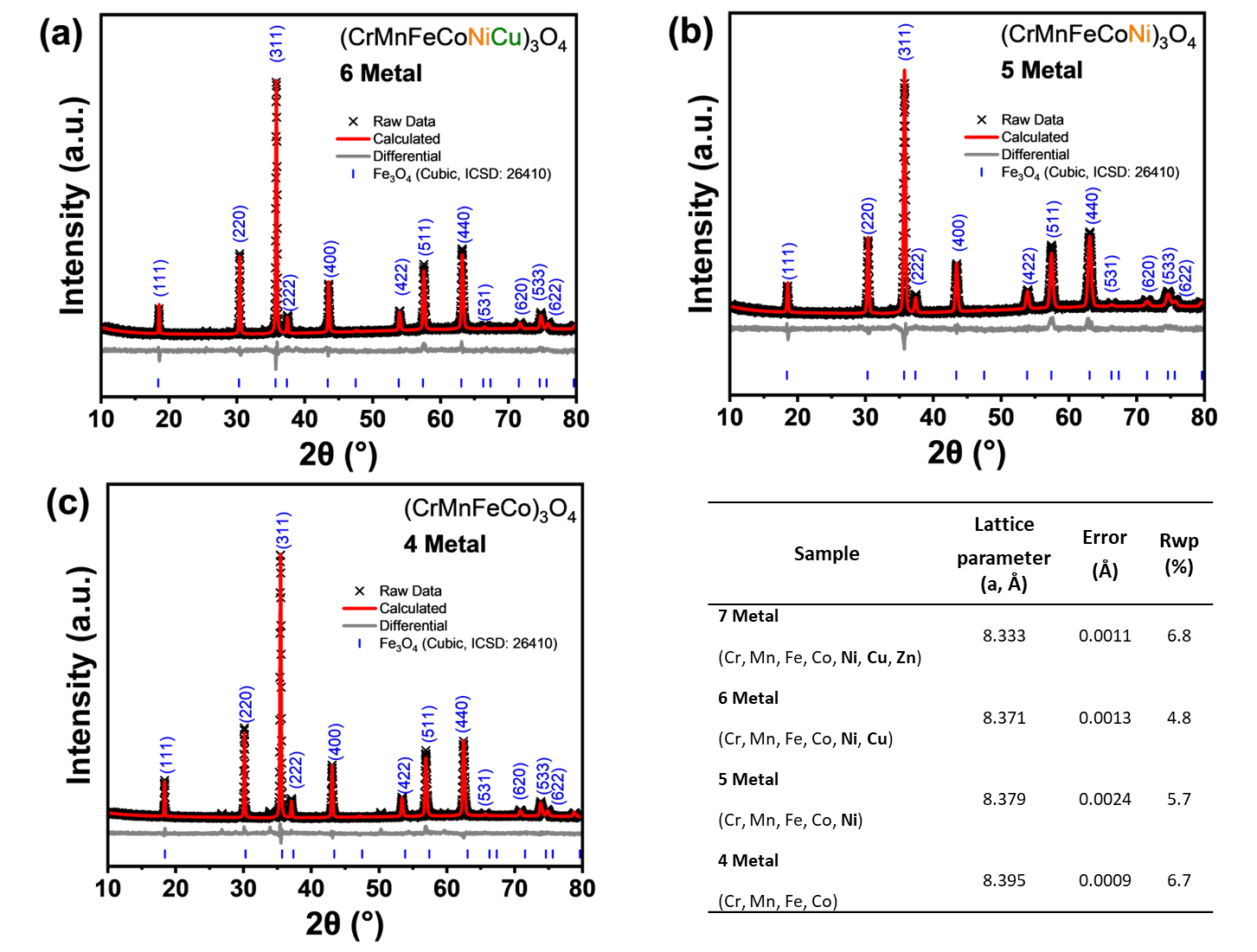


**Figure S10.** (a) – (c) Rietveld refinement of 6, 5, and 4 metal HESO powders respectively (d) Calculated lattice parameter, the associated parameter error, and a measure of the quality of fit via the Rietveld refinement parameter (Rwp) are summarized for all samples including the 7 Metal HESO.


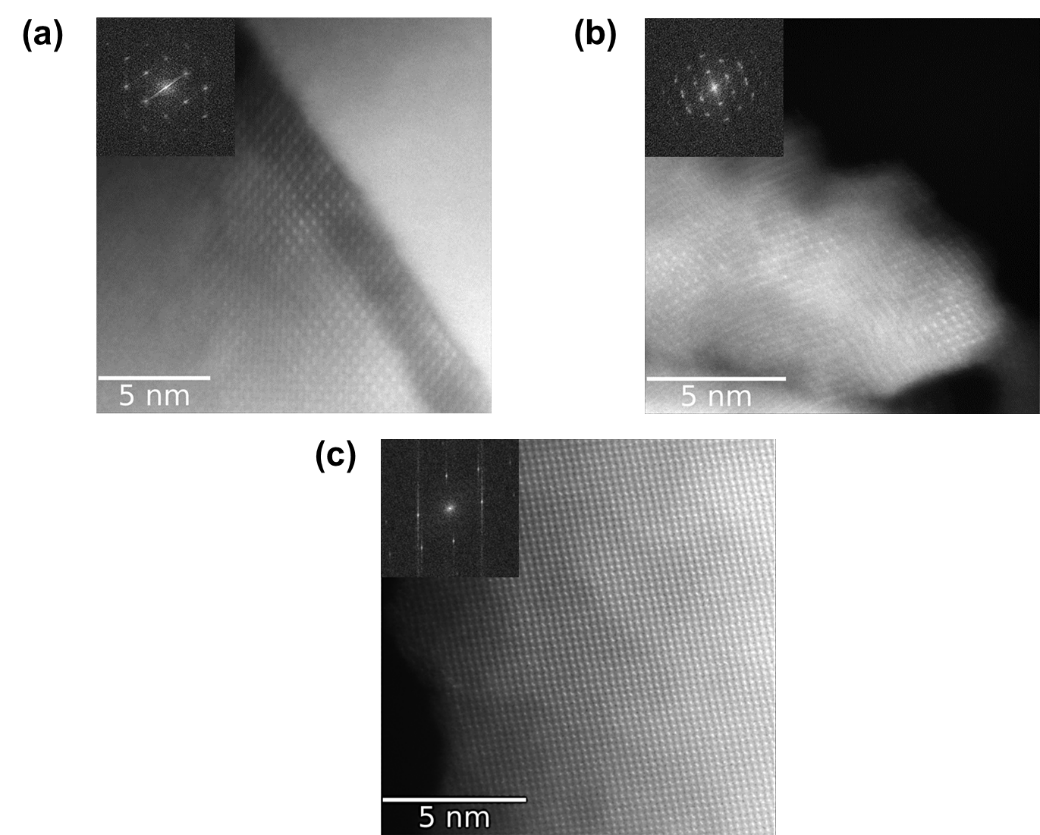


**Figure S11.** Atomic resolution HAADF-STEM images with Fast Fourier Transform (FFT) insets for (a) 6-metal, (b) 5-metal, and (c) 4-metal HESOs. The zone axis shown in the images for the 6 and 5-metal is the [110], whereas the zone axis for the 4-metal was the [311] (all indexed to the expected spinel crystal structure).


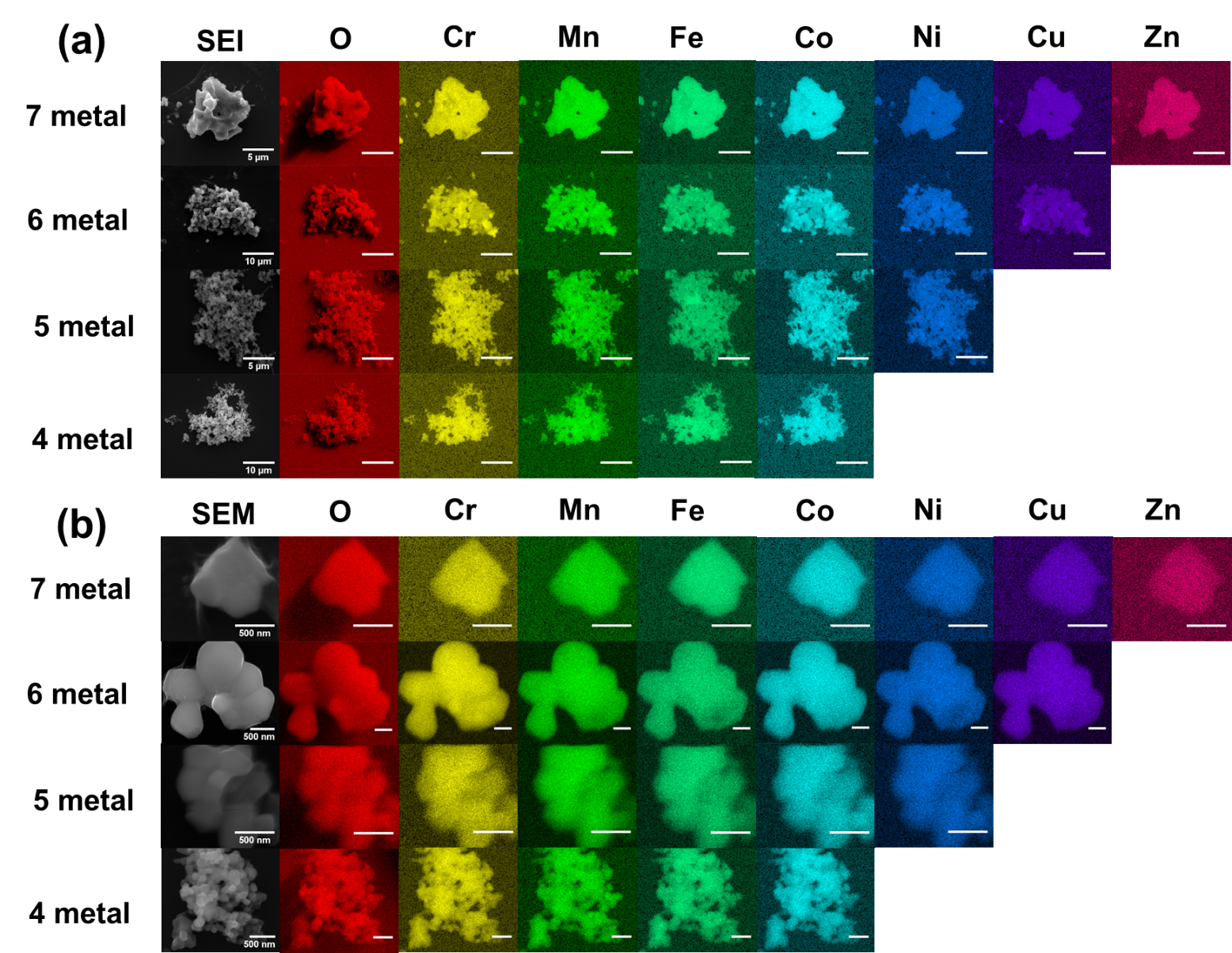


**Figure S12.** (a) SEM-EDS (20 kV) of all 5 HESO samples including a secondary electron image (SEI) then O, and various metallic elemental distribution maps at both (a) micro and (b) nanoscale. Scale bars represent either 5 or 10 μm for (a) and 500 nm for (b). Due to high associated errors with these values (e.g., unquantifiability of oxygen, overlap of K-edge of some metals are not resolvable with SEM-EDS and sample preparation on glass slide) no quantitative atomic percentages are provided. No detectable sulfur was present, as highlighted in the EDS spectra below.


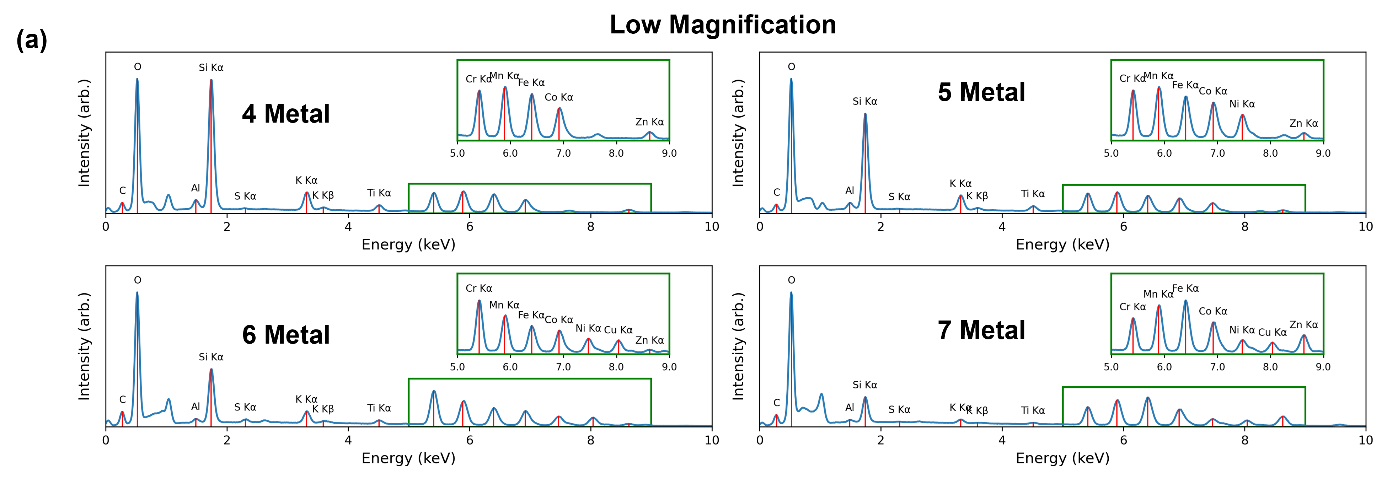

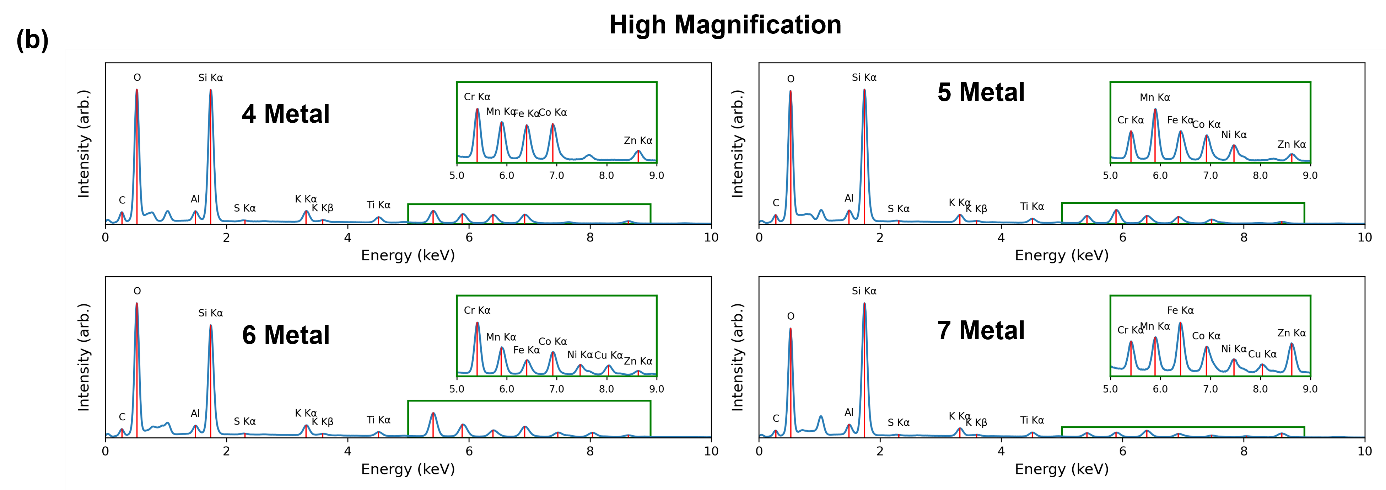


**Figure S13.** SEM**-**EDS spectra for 4 to 7 metal HESO powders at (a) low and (b) high magnification for SEM-EDS maps from Figure S12. The samples are prepared on glass slides to prevent acquisition issues associated with the magnetic properties of spinels. The peaks associated with Ti, Si, K, and Zn are from the glass slide, and the Al is associated with the SEM stub. Therefore, quantification is not feasible with SEM-EDS and only qualitative conclusions of elemental distribution can be made.


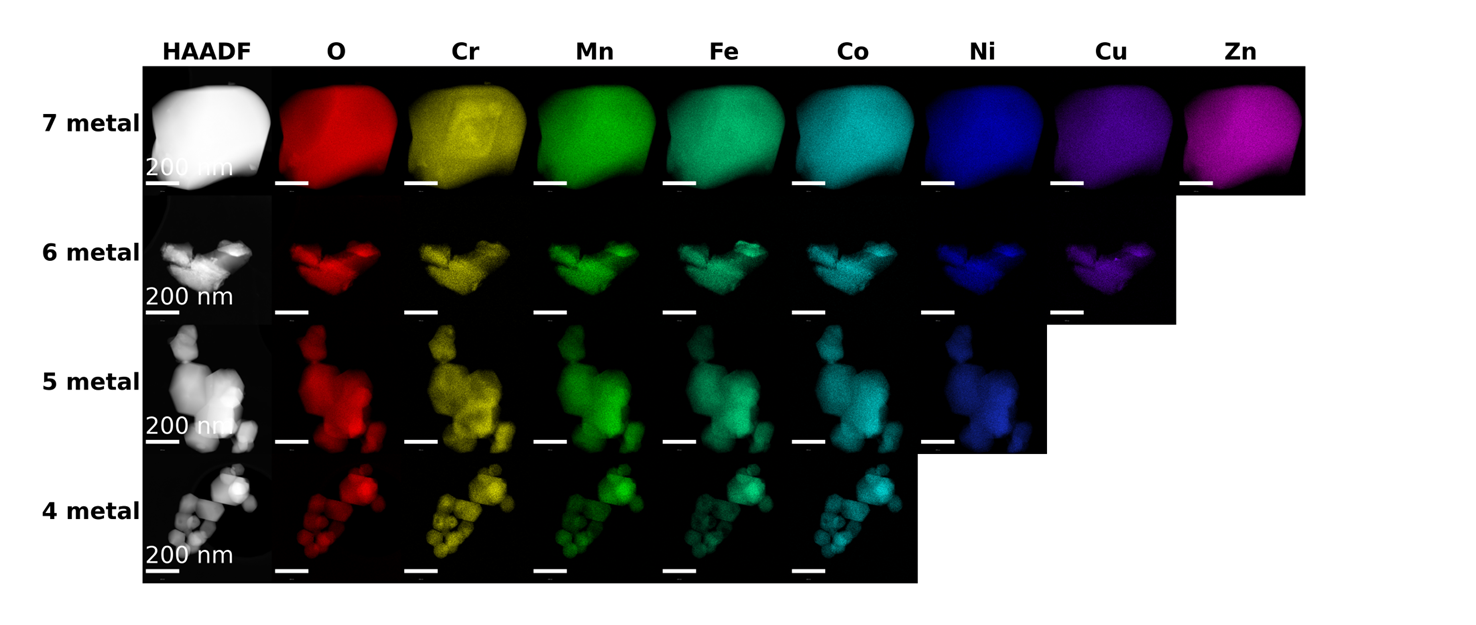


**Figure S14.** STEM-HAADF images and EDS (200 kV) mapping at a lower magnification for all 4 to 7 metal samples


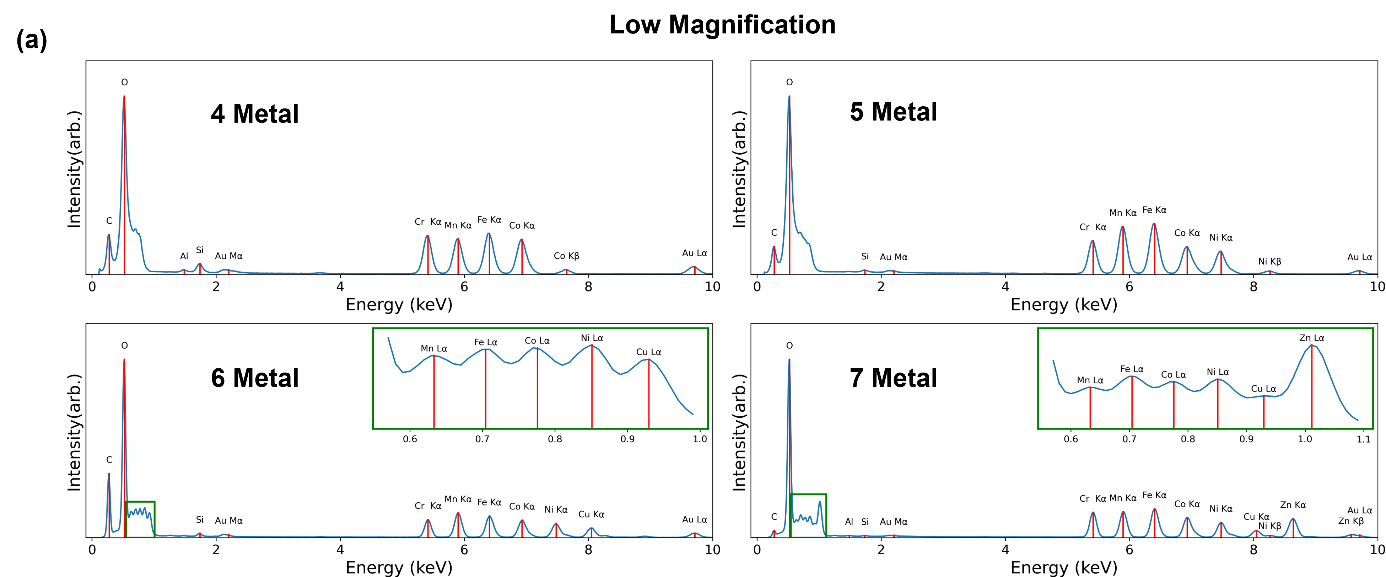

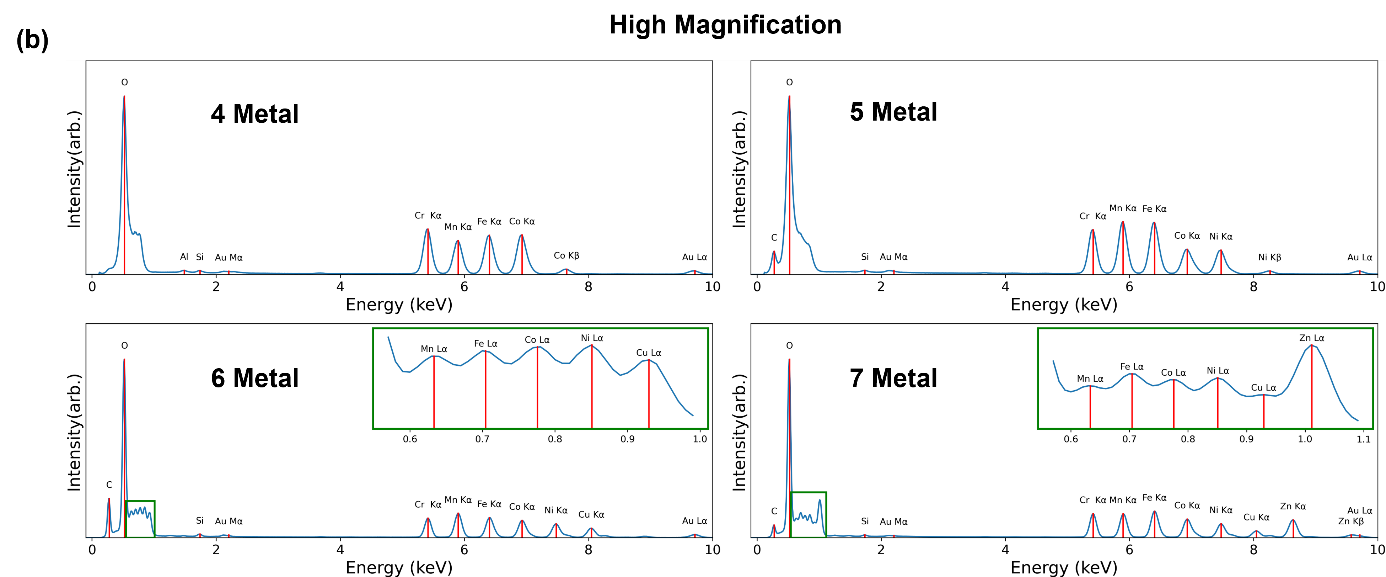


**Figure S15.** STEM-EDS spectra for 4 to 7 at (a) low and (b) high magnification for EDS maps from Figure S14 and Figure 5, respectively.

**Table S3.** (a) Averaged atomic percentages from 5 different STEM-EDS maps (not shown) of each HESO powder with estimated errors. (b) the estimated composition calculated from (a) compared to target compositions.

**(a)**

| **Sample** | **O /At.%** | **S /At.%** | **Cr /At.%** | **Mn /At.%** | **Fe /At.%** | **Co /At.%** | **Ni /At.%** | **Cu /At.%** | **Zn /At.%** |
| --- | --- | --- | --- | --- | --- | --- | --- | --- | --- |
| **7 metal**  (Cr, Mn, Fe, Co, **Ni**, **Cu**, **Zn**) | 64.5 ± 2.4 | 0 | 4.9 ± 0.9 | 5.8 ± 0.9 | 6.5 ± 0.9 | 5.4 ± 1.0 | 3.6 ± 0.6 | 2.5 ± 0.3 | 4.7 ± 0.8 |
| **6 metal**  (Cr, Mn, Fe, Co, **Ni**, **Cu**) | 68.3 ± 1.5 | 0.2 ± 0.1 | 7.9 ± 1.1 | 5.4 ± 0.8 | 5.4 ± 0.7 | 5.7 ± 0.7 | 3.5 ± 0.5 | 3.6 ± 0.5 |  |
| **5 metal**  (Cr, Mn, Fe, Co, **Ni**) | 51.4 ± 1.7 | 0 | 9.7 ± 1.2 | 10.5 ± 1.5 | 10.3 ± 1.3 | 10.0 ± 1.3 | 8.1 ± 1.1 |  |  |
| **4 metal**  (Cr, Mn, Fe, Co) | 52.9 ± 1.8 | 0 | 11.7 ± 1.4 | 11.9 ± 1.7 | 13.1 ± 1.6 | 10.5 ± 1.3 |  |  |  |

**(b)**

| **Sample** | **Target composition** | **Composition by STEM-EDS** | **Composition by XPS** |
| --- | --- | --- | --- |
| **7 metal**  (Cr, Mn, Fe, Co, **Ni**, **Cu**, **Zn**) | (Cr_0.14_ Mn_0.14_ Fe_0.14_ Co_0.14_ **Ni_0.14_ Cu_0.14_ Zn_0.14_**)_3_O_4_ | (Cr_0.14_ Mn_0.16_ Fe_0.18_ Co_0.15_ **Ni_0.10_ Cu_0.07_ Zn_0.13_**)_3_O_4_ | (Cr_0.24_ Mn_0.17_ Fe_0.21_ Co_0.15_ **Ni_0.07_ Cu_0.09_ Zn_0.08_**)_3_O_4_ |
| **6 metal**  (Cr, Mn, Fe, Co, **Ni**, **Cu**) | (Cr_0.17_ Mn_0.17_ Fe_0.17_ Co_0.17_ **Ni_0.17_ Cu_0.17_**)_3_O_4_ | (Cr_0.25_ Mn_0.17_ Fe_0.17_ Co_0.18_ **Ni_0.11_ Cu_0.11_**)_3_O_4_ | (Cr_0.19_ Mn_0.27_ Fe_0.17_ Co_0.20_ **Ni_0.07_ Cu_0.11_**)_3_O_4_ |
| **5 metal**  (Cr, Mn, Fe, Co, **Ni**) | (Cr_0.20_ Mn_0.20_ Fe_0.20_ Co_0.20_ **Ni_0.20_**)_3_O_4_ | (Cr_0.20_ Mn_0.22_ Fe_0.21_ Co_0.21_ **Ni_0.17_**)_3_O_4_ | (Cr_0.24_ Mn_0.32_ Fe_0.17_ Co_0.17_ **Ni_0.10_**)_3_O_4_ |
| **4 metal**  (Cr, Mn, Fe, Co) | (Cr_0.25_ Mn_0.25_ Fe_0.25_ Co_0.25_)_3_O_4_ | (Cr_0.25_ Mn_0.25_ Fe_0.28_ Co_0.22_)_3_O_4_ | (Cr_0.26_ Mn_0.32_ Fe_0.24_ Co_0.19_)_3_O_4_ |


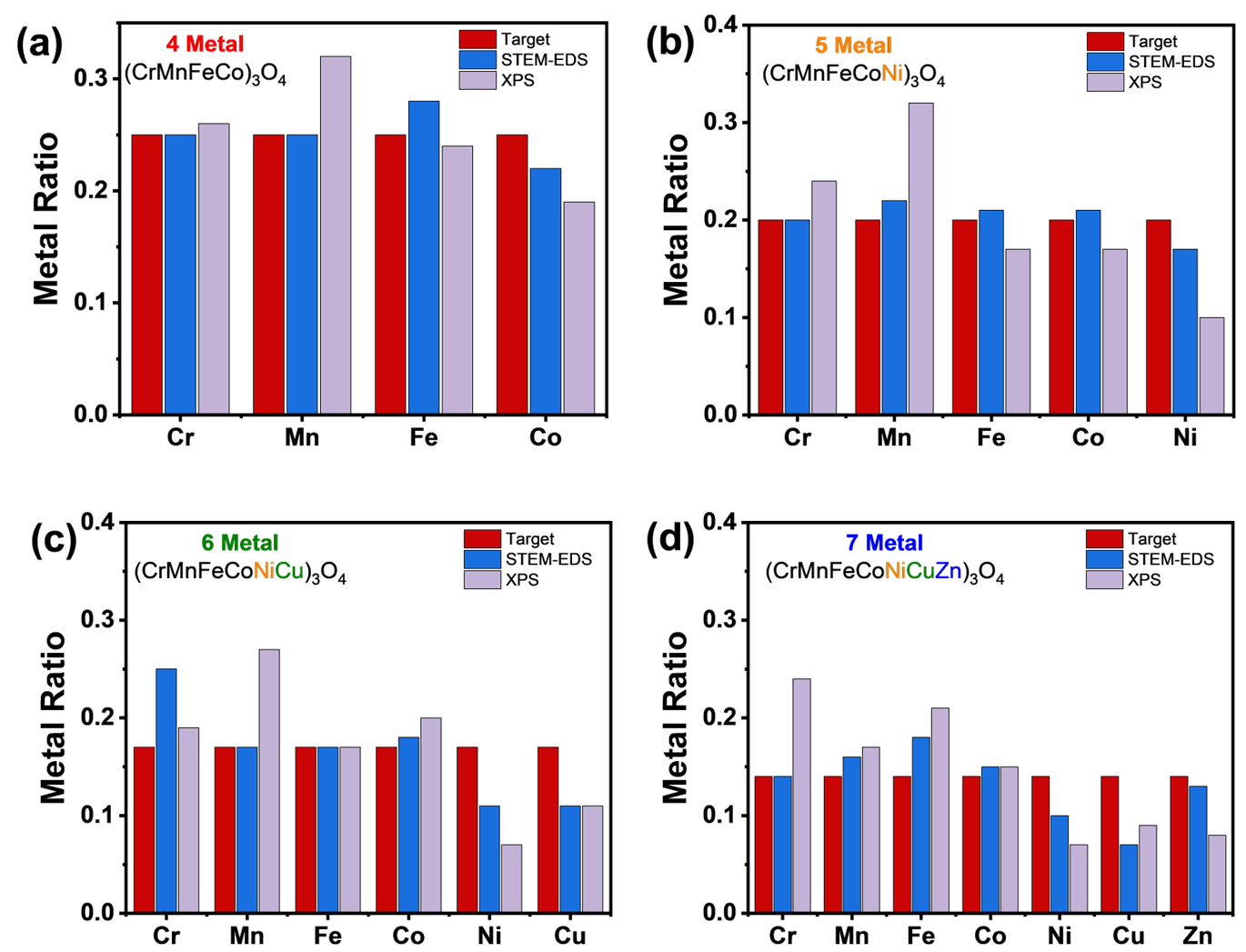


### **Figure S16.** Graphic comparison of the changing metal ratios between target, STEM-EDS, and XPS calculated compositions for 4-, 5-, 6-, and 7-metal HESOs (a)-(d) respectively.


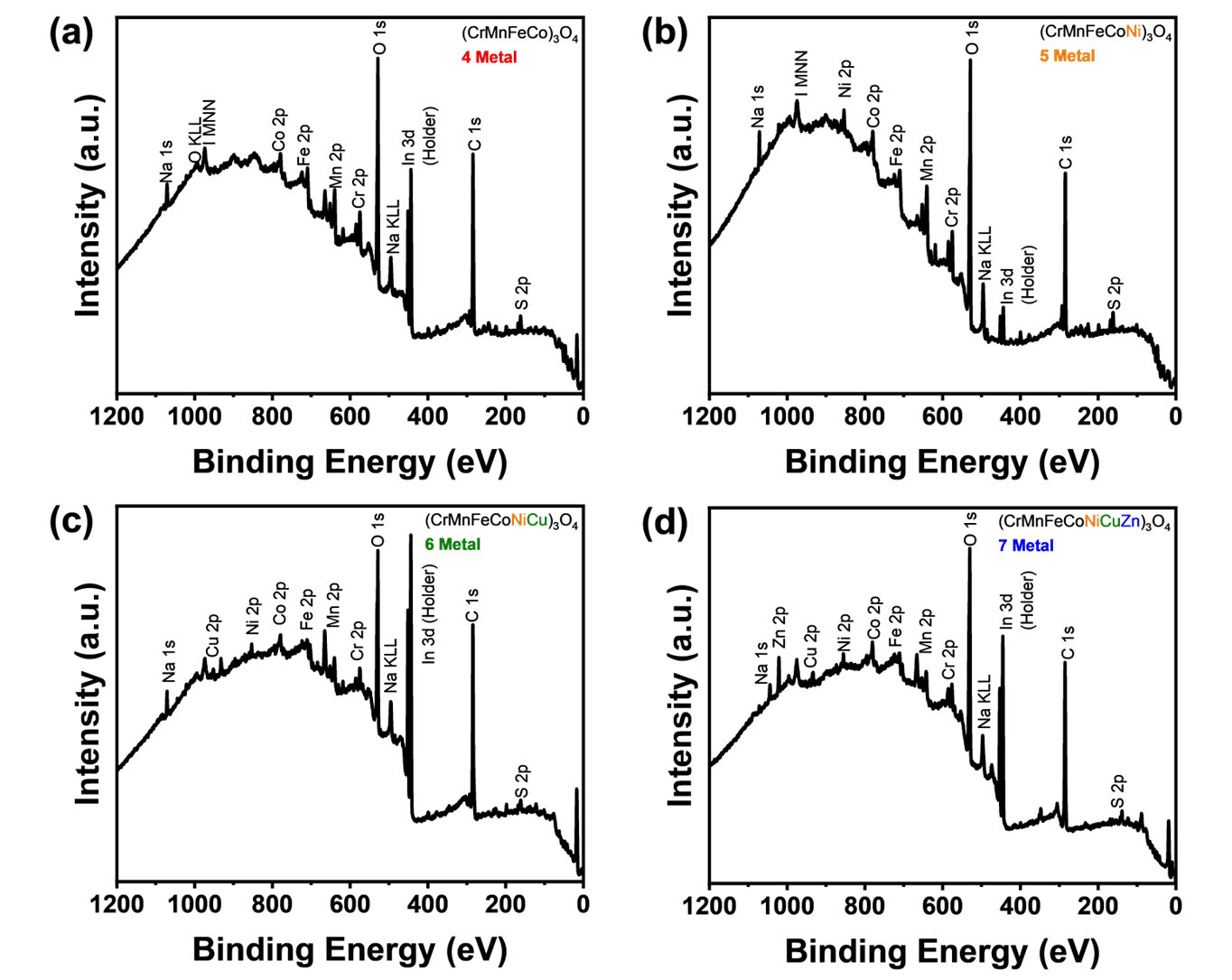


**Figure S17.** XPS survey spectra of 4-metal, 5-metal, 6-metal, and 7-metal HESO powders (a)-(d) respectively.


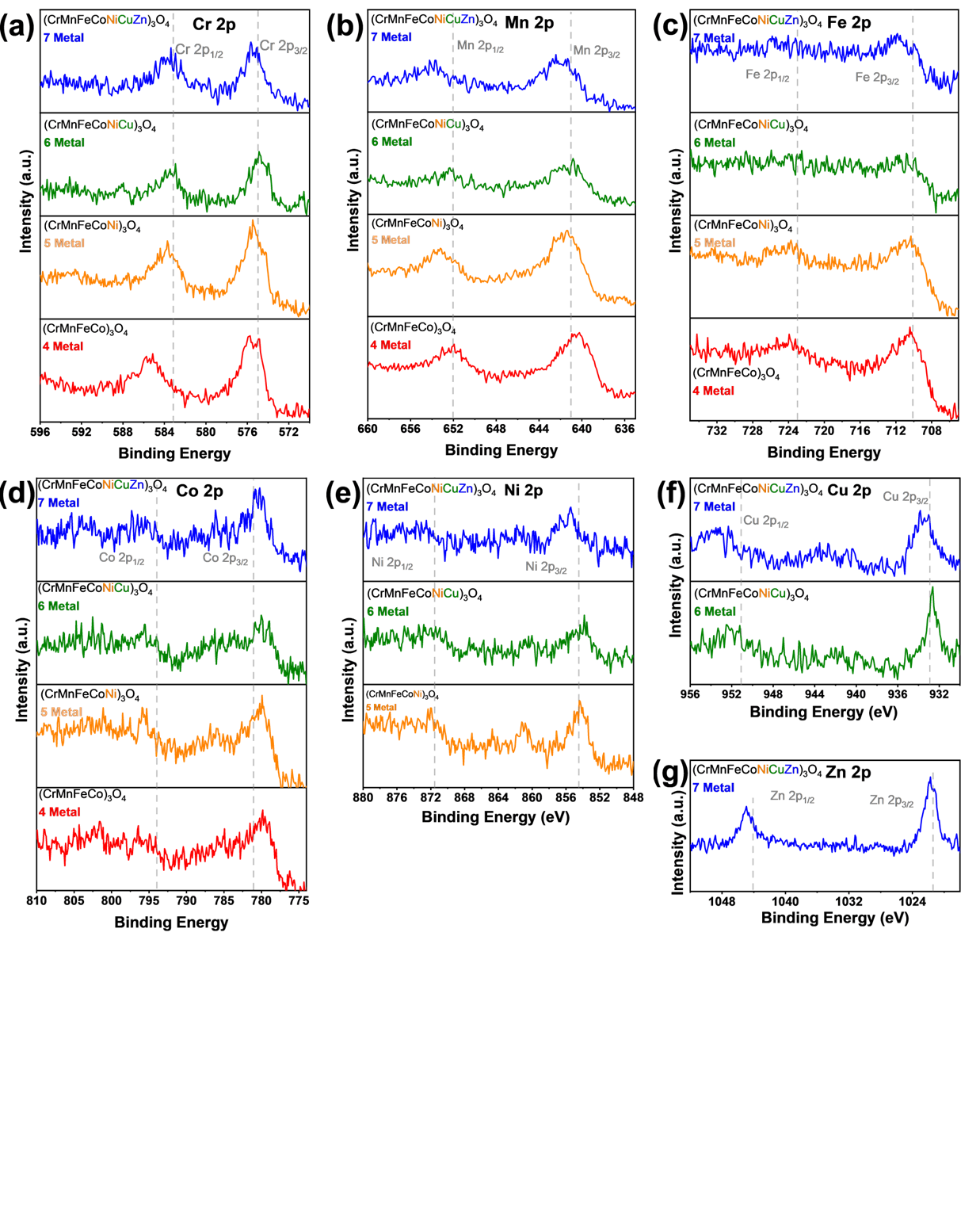


**Figure S18.** High-resolution XPS spectra of (a) Cr, (b) Mn, (c) Fe, (d) Co, (e) Ni, (f) Cu, and (g) Zn 2p core levels for 4-, 5-, 6-, and 7-metal HESO powders.


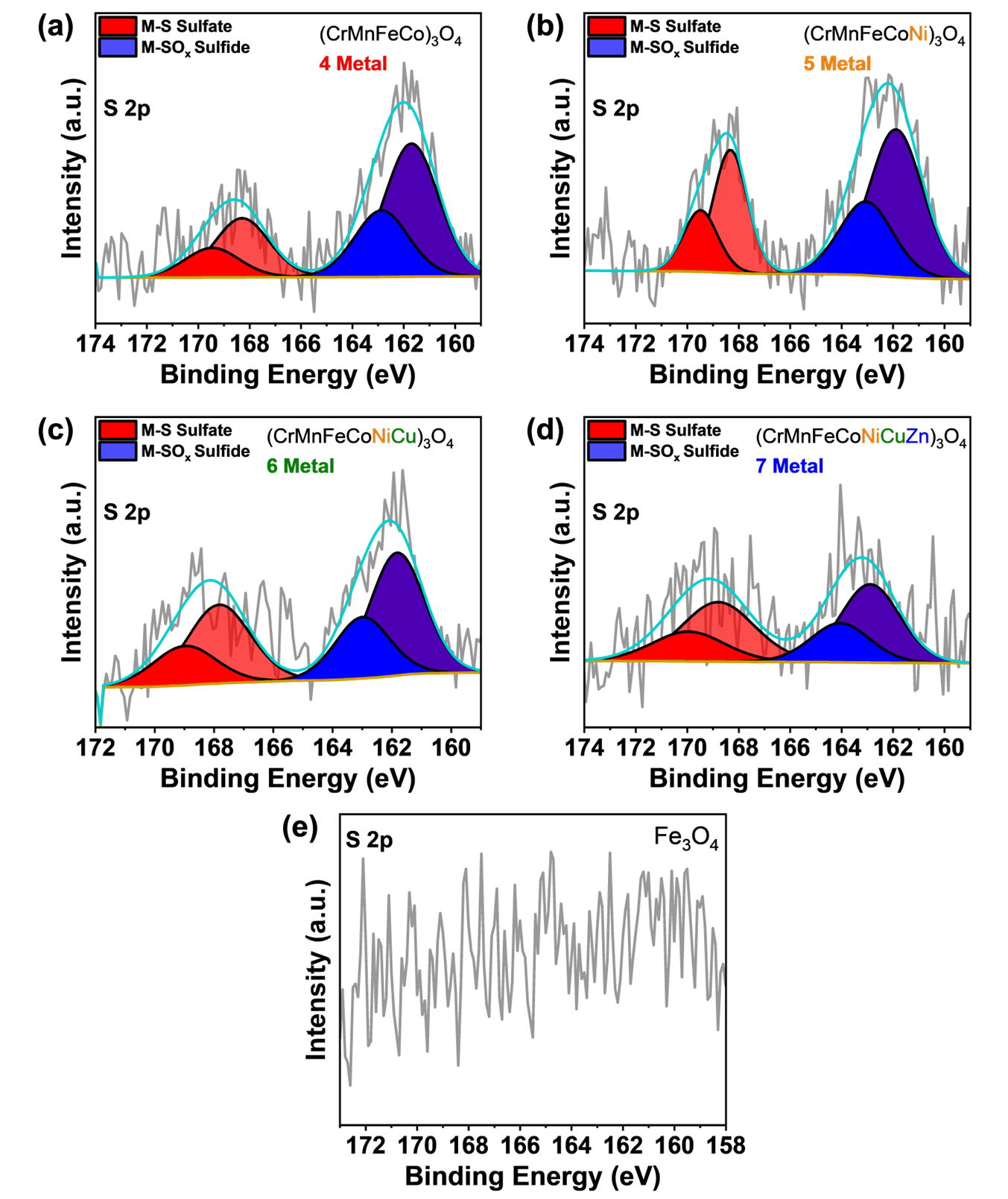


**Figure S19.** (a)-(d) High-resolution XPS spectra of S 2p core level for 4-metal, 5-metal, 6-metal, and 7-metal HESO powders. Two species were identified as associated with sulfate and sulfide. (e) High-resolution XPS spectra of the commercial Fe_3_O_4_ shows no similar sulfur species.

**Table S4.** Element quantification of 4-metal, 5-metal, 6-metal, and 7-metal HESO powders from XPS spectra. (a) Metal to oxygen ratio only (b) transition metals, oxygen, and sulfur at.%. It should be noted that quantification of the O1s peak included other surface organic oxygen species which significantly increases the O 1s peak area and reduces the oxygen-to-metal ratio from the ideal 0.75. Qualitatively, the metal-to-oxygen ratio does however remain similar between different samples. Overlapping Auger peaks from several transition metals contained within each sample can also account for some of the detected variation between different metal at.%

**(a) Metals and Oxygen Only**

| **Sample** | **O /At.%** | **Cr /At.%** | **Mn /At.%** | **Fe /At.%** | **Co /At.%** | **Ni /At.%** | **Cu /At.%** | **Zn /At.%** | **Oxygen: Metal**  **Ratio** |
| --- | --- | --- | --- | --- | --- | --- | --- | --- | --- |
| **7 Metal**  (Cr, Mn, Fe, Co, **Ni**, **Cu**, **Zn**) | 81.1 ± 0.7 | 4.5 ± 0.4 | 3.2 ± 0.4 | 3.9 ± 0.4 | 2.9 ± 0.3 | 1.3 ± 0.3 | 1.6 ± 0.3 | 1.5 ± 0.1 | 0.23 |
| **6 Metal**  (Cr, Mn, Fe, Co, **Ni**, **Cu**) | 82.1 ± 0.9 | 3.4 ± 0.4 | 4.8 ± 0.5 | 3.1 ± 0.4 | 3.5 ± 0.4 | 1.3 ± 0.3 | 2.0 ± 0.3 |  | 0.22 |
| **5 Metal**  (Cr, Mn, Fe, Co, **Ni**) | 77.7 ± 0.7 | 5.4 ± 0.3 | 7.2 ± 0.4 | 3.7 ± 0.5 | 3.8 ± 0.4 | 2.2 ± 0.3 |  |  | 0.29 |
| **4 Metal**  (Cr, Mn, Fe, Co) | 80.0 ± 0.8 | 5.2 ± 0.4 | 6.3 ± 0.5 | 4.8 ± 0.5 | 3.8 ± 0.3 |  |  |  | 0.25 |

**(b) Metals, Sulfur and Oxygen**

| **Sample** | **O /At.%** | **S /At.%** | **Cr /At.%** | **Mn /At.%** | **Fe /At.%** | **Co /At.%** | **Ni /At.%** | **Cu /At.%** | **Zn /At.%** |
| --- | --- | --- | --- | --- | --- | --- | --- | --- | --- |
| **7 Metal**  (Cr, Mn, Fe, Co, **Ni**, **Cu**, **Zn**) | 76.7 ± 1.1 | 5.4 ± 1.1 | 4.2 ± 0.4 | 3.0 ± 0.4 | 3.7 ± 0.4 | 2.8 ± 0.3 | 1.3 ± 0.3 | 1.5 ± 0.2 | 1.4 ± 0.1 |
| **6 Metal**  (Cr, Mn, Fe, Co, **Ni**, **Cu**) | 72.5 ± 1.1 | 11.8 ± 1.0 | 3.0 ± 0.4 | 4.2 ± 0.5 | 2.7 ± 0.4 | 3.1 ± 0.4 | 1.1 ± 0.3 | 1.7 ± 0.3 |  |
| **5 Metal**  (Cr, Mn, Fe, Co, **Ni**) | 67.6 ± 0.8 | 12.9 ± 0.8 | 4.7 ± 0.3 | 6.3 ± 0.3 | 3.3 ± 0.4 | 3.3 ± 0.3 | 1.9 ± 0.2 |  |  |
| **4 Metal**  (Cr, Mn, Fe, Co) | 69.0 ± 1.0 | 13.7 ± 1.0 | 4.5 ± 0.3 | 5.4 ± 0.5 | 4.2 ± 0.5 | 3.2 ± 0.3 |  |  |  |


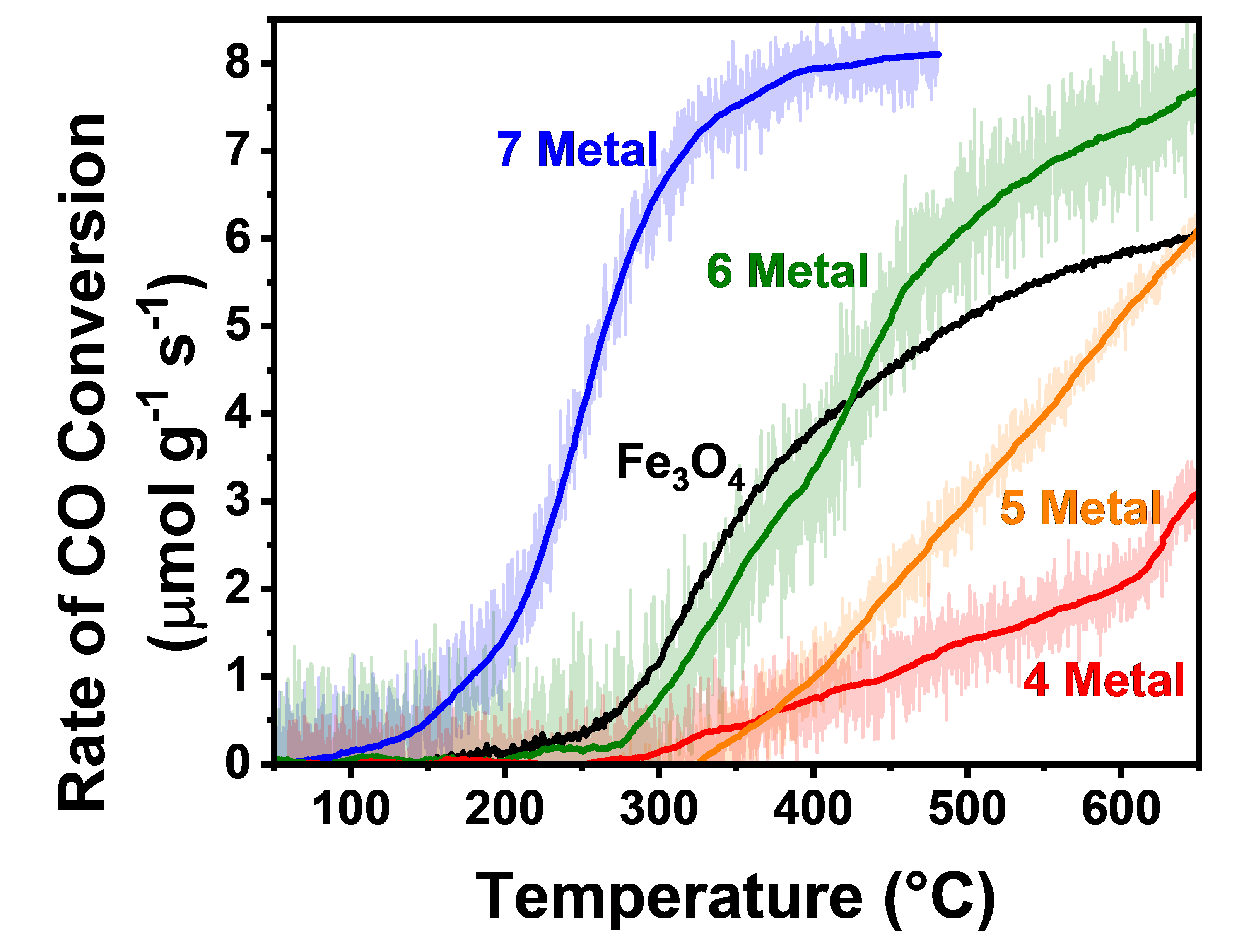


### **Figure S20.** The rate of CO conversion per gram of catalyst (μmol g^-1^ s^-1^) as a function of temperature to account for differences in mass loading. This shows that the trend is maintained when independent of mass loading.


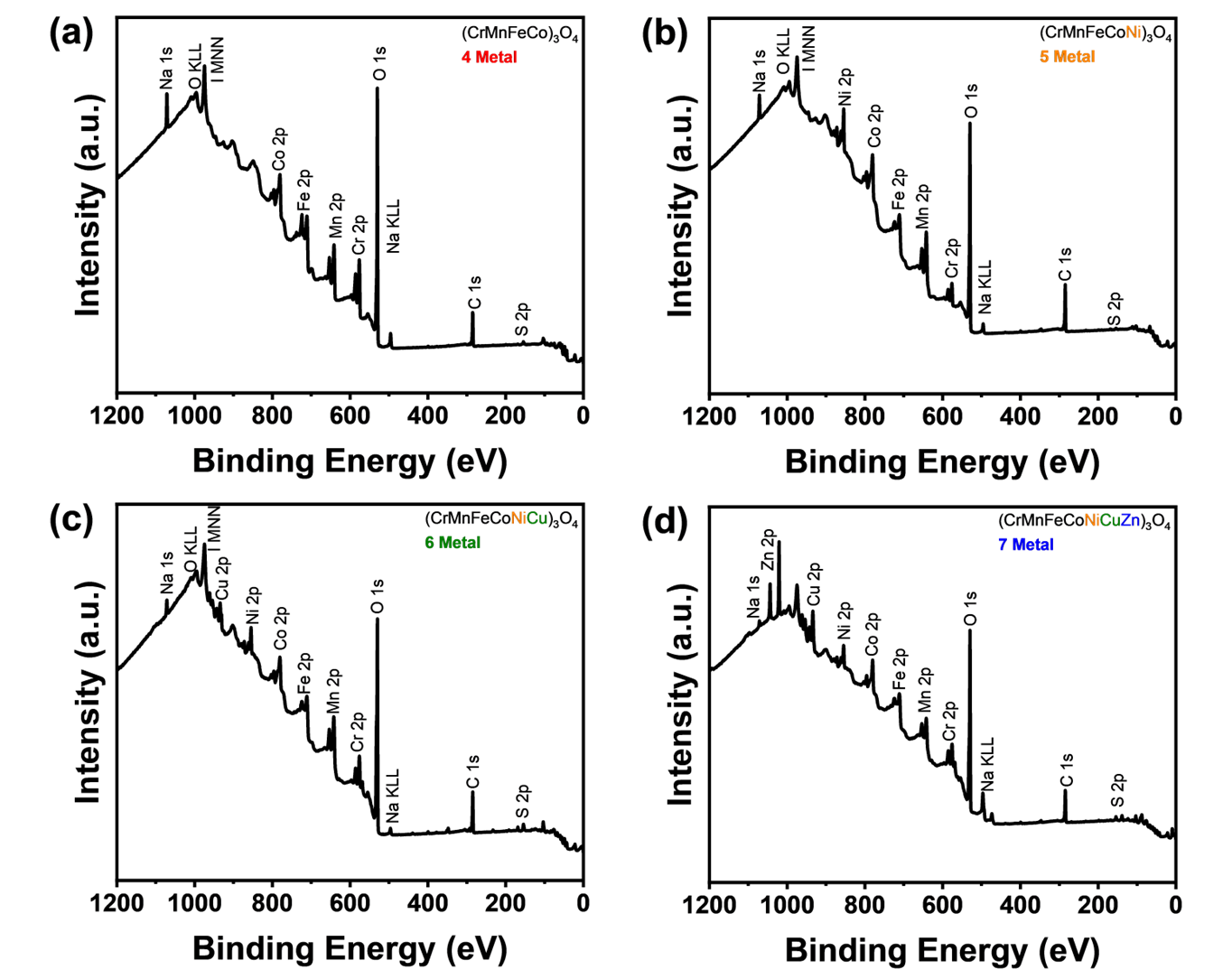


**Figure S21.** (a)-(d) Survey spectra of the 4-metal, 5-metal, 6-metal, and 7-metal HESO powders post catalysis.

**Table S5.** Elemental quantification (at.%) of samples post-catalysis using XPS spectra without (a) and (b) including sulfur. (c) shows the estimated surface composition post-catalysis compared to the synthesized surface compositions.

**(a) Metals and Oxygen Only**

| **Sample** | **O /At.%** | **Cr /At.%** | **Mn /At.%** | **Fe /At.%** | **Co /At.%** | **Ni /At.%** | **Cu /At.%** | **Zn /At.%** | **Metal:**  **Oxygen**  **Ratio** |
| --- | --- | --- | --- | --- | --- | --- | --- | --- | --- |
| **7 Metal**  (Cr, Mn, Fe, Co, **Ni**, **Cu**, **Zn**) | 65.6 ± 0.1 | 4.4 ± 0.1 | 6.9 ± 0.1 | 6.3 ± 0.1 | 5.3 ± 0.1 | 3.0 ± 0.1 | 5.3 ± 0.1 | 3.3 ± 0.1 | 0.52 |
| **6 Metal**  (Cr, Mn, Fe, Co, **Ni**, **Cu**) | 71.7 ± 0.1 | 3.7 ± 0.1 | 8.6 ± 0.1 | 5.8 ± 0.1 | 5.5 ± 0.1 | 2.5 ± 0.1 | 2.4 ± 0.1 |  | 0.39 |
| **5 Metal**  (Cr, Mn, Fe, Co, **Ni**) | 66.7 ± 0.1 | 3.5 ± 0.1 | 9.8 ± 0.1 | 6.5 ± 0.1 | 8.5 ± 0.1 | 5.0 ± 0.1 |  |  | 0.50 |
| **4 Metal**  (Cr, Mn, Fe, Co) | 67.9 ± 0.8 | 8.4 ± 0.1 | 7.6 ± 0.1 | 8.5 ± 0.1 | 7.7 ± 0.1 |  |  |  | 0.47 |

**(b) Metals, Sulfur and Oxygen**

| **Sample** | **O /At.%** | **S /At.%** | **Cr /At.%** | **Mn /At.%** | **Fe /At.%** | **Co /At.%** | **Ni /At.%** | **Cu /At.%** | **Zn /At.%** |
| --- | --- | --- | --- | --- | --- | --- | --- | --- | --- |
| **7 Metal**  (Cr, Mn, Fe, Co, **Ni**, **Cu**, **Zn**) | 65.3 ± 0.1 | 0.3 ± 0.1 | 4.4 ± 0.1 | 6.8 ± 0.1 | 6.3 ± 0.1 | 5.3 ± 0.1 | 3.0 ± 0.1 | 5.3 ± 0.1 | 3.3 ± 0.1 |
| **6 Metal**  (Cr, Mn, Fe, Co, **Ni**, **Cu**) | 70.4 ± 0.1 | 1.9 ± 0.1 | 3.6 ± 0.1 | 8.3 ± 0.1 | 5.7 ± 0.1 | 5.4 ± 0.1 | 2.4 ± 0.1 | 2.3 ± 0.1 |  |
| **5 Metal**  (Cr, Mn, Fe, Co, **Ni**) | 66.5 ± 0.1 | 0.3 ± 0.1 | 3.5 ± 0.1 | 9.8 ± 0.1 | 6.5 ± 0.1 | 8.5 ± 0.1 | 5.0 ± 0.1 |  |  |
| **4 Metal**  (Cr, Mn, Fe, Co) | 67.7 ± 0.1 | 0.3 ± 0.1 | 8.4 ± 0.1 | 7.5 ± 0.1 | 8.5 ± 0.1 | 7.7 ± 0.1 |  |  |  |

**(c)**

| **Sample** | **Target composition** | Composition by XPS  **Pre-catalysis** | Composition by XPS  **Post-catalysis** |
| --- | --- | --- | --- |
| **7 metal**  (Cr, Mn, Fe, Co, **Ni**, **Cu**, **Zn**) | (Cr_0.14_ Mn_0.14_ Fe_0.14_ Co_0.14_ **Ni_0.14_ Cu_0.14_ Zn_0.14_**)_3_O_4_ | (Cr_0.24_ Mn_0.17_ Fe_0.21_ Co_0.15_ **Ni_0.07_ Cu_0.09_ Zn_0.08_**)_3_O_4_ | (Cr_0.13_ Mn_0.20_ Fe_0.18_ Co_0.15_ **Ni_0.09_ Cu_0.15_ Zn_0.10_**)_3_O_4_ |
| **6 metal**  (Cr, Mn, Fe, Co, **Ni**, **Cu**) | (Cr_0.17_ Mn_0.17_ Fe_0.17_ Co_0.17_ **Ni_0.17_ Cu_0.17_**)_3_O_4_ | (Cr_0.19_ Mn_0.27_ Fe_0.17_ Co_0.20_ **Ni_0.07_ Cu_0.11_**)_3_O_4_ | (Cr_0.13_ Mn_0.30_ Fe_0.20_ Co_0.19_ **Ni_0.09_ Cu_0.08_**)_3_O_4_ |
| **5 metal**  (Cr, Mn, Fe, Co, **Ni**) | (Cr_0.20_ Mn_0.20_ Fe_0.20_ Co_0.20_ **Ni_0.20_**)_3_O_4_ | (Cr_0.24_ Mn_0.32_ Fe_0.17_ Co_0.17_ **Ni_0.10_**)_3_O_4_ | (Cr_0.11_ Mn_0.29_ Fe_0.20_ Co_0.26_ **Ni_0.15_**)_3_O_4_ |
| **4 metal**  (Cr, Mn, Fe, Co) | (Cr_0.25_ Mn_0.25_ Fe_0.25_ Co_0.25_)_3_O_4_ | (Cr_0.26_ Mn_0.32_ Fe_0.24_ Co_0.19_)_3_O_4_ | (Cr_0.26_ Mn_0.24_ Fe_0.26_ Co_0.24_)_3_O_4_ |


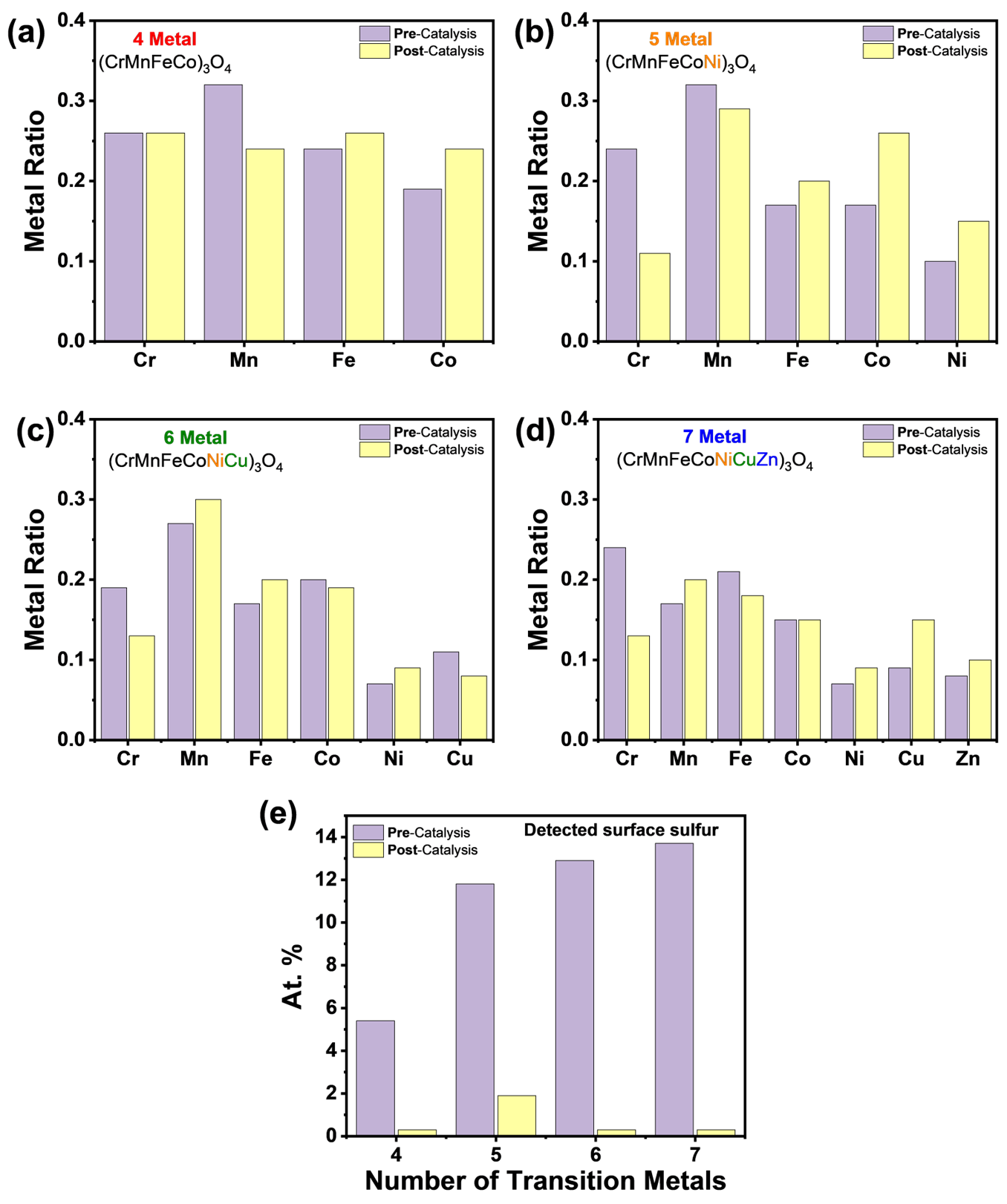


### **Figure S22.** Graphic comparison of the changing metal ratios between pre-catalysis and post-catalysis as calculated by XPS for 4-, 5-, 6-, and 7-metal HESOs (a)-(d) respectively. (e) shows the changing at.% of sulfur pre- and post-catalysis.


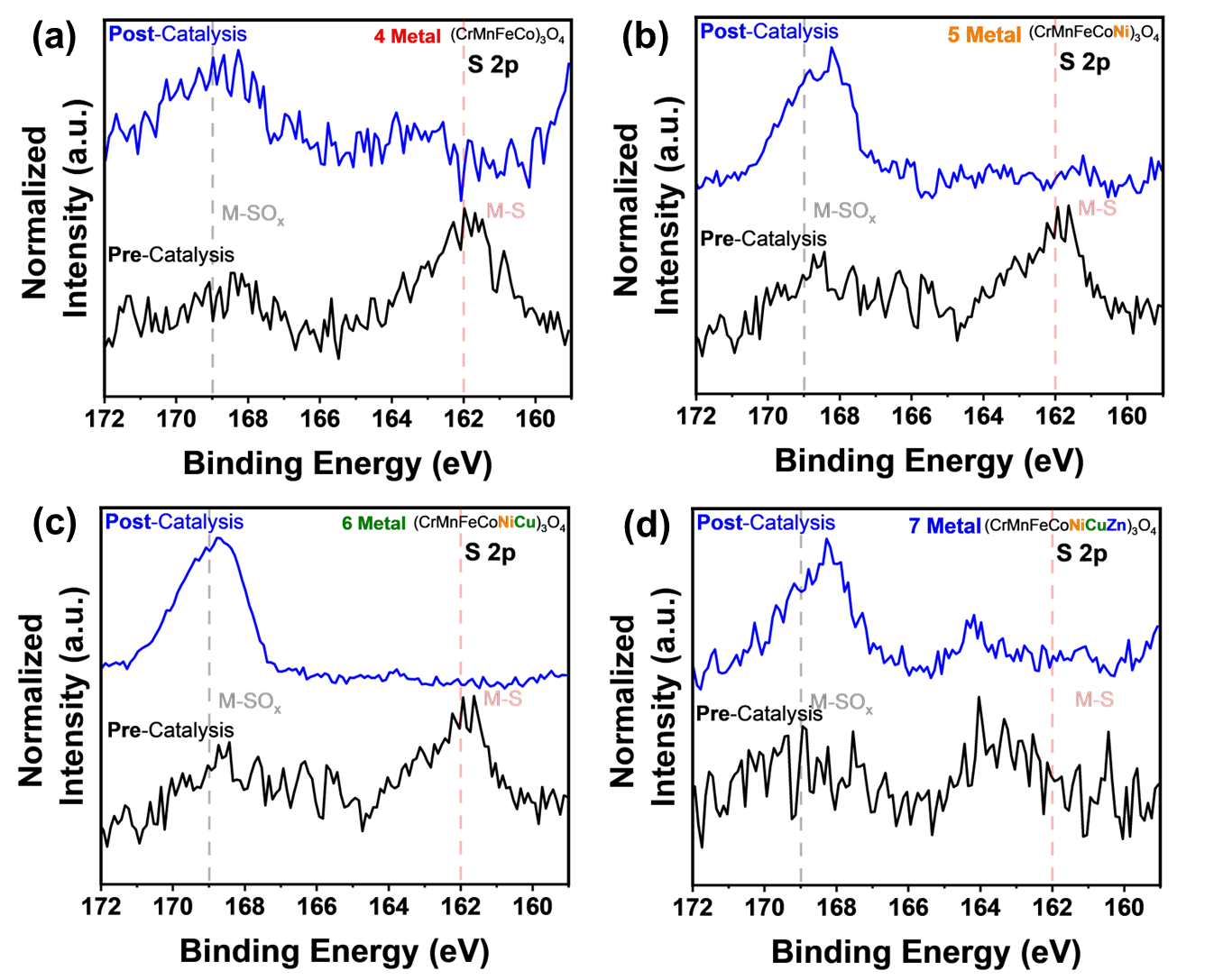


**Figure S23.** (a)-(d) Comparison of the high-resolution S 2p spectra of the 4-metal, 5-metal, 6-metal, and 7-metal HESO powders post catalysis. Spectra are normalized to the most significant peak to allow sufficient comparison between the sulfate and sulfide species.


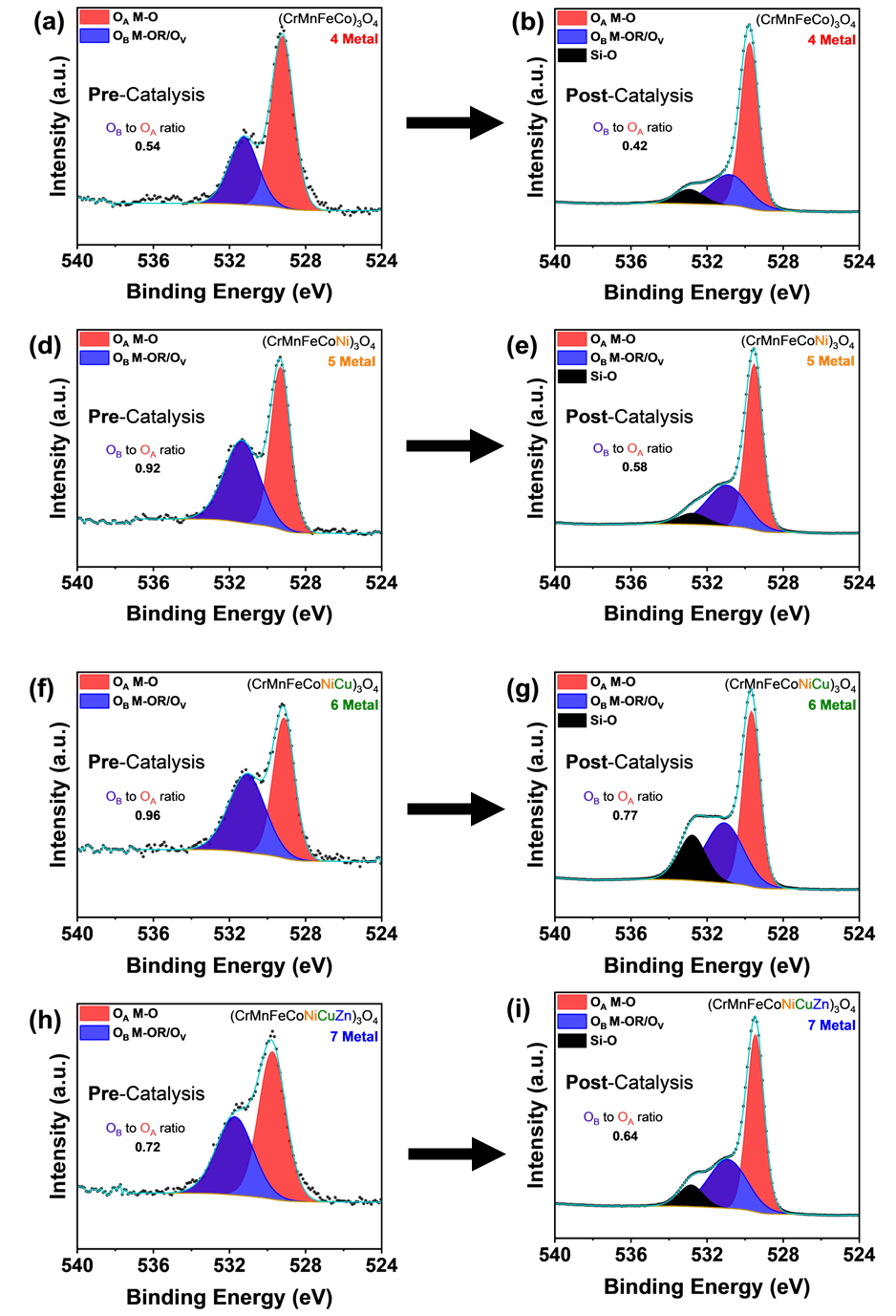


**Figure S24.** (a)-(i) Comparison of the high-resolution O 1s spectra of the 4-metal, 5-metal, 6-metal, and 7-metal HESO powders pre- (left-hand side) and post (right-hand side) catalysis. O_B_-to-O_A_ ratios are provided to quantitatively assess the changes between these different species. The plug flow setup uses quartz wool to create a reactor bed, leaving some surface Si-O groups at high binding energy post-catalysis.


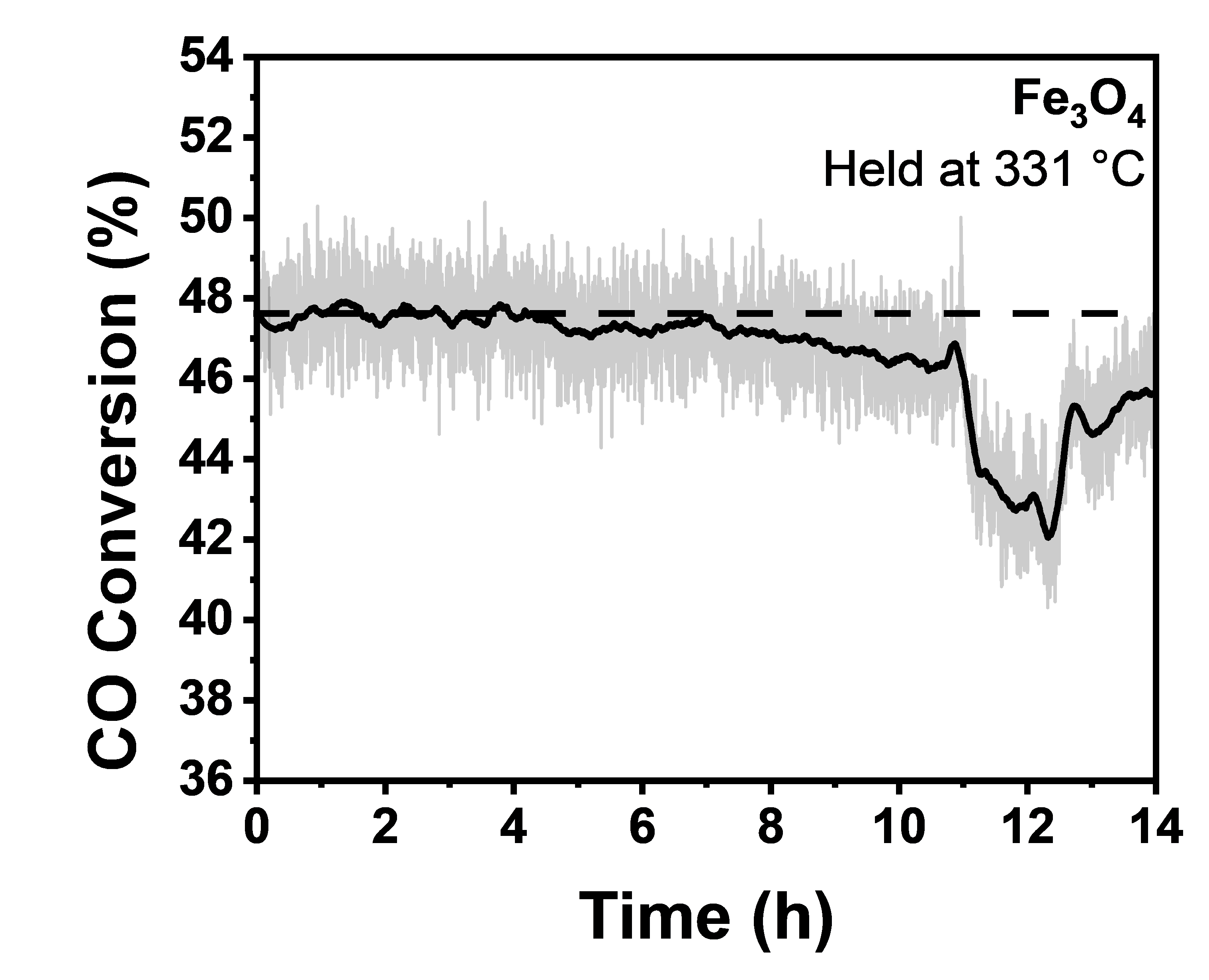


**Figure S25.** Long-term catalyst stability test of commercial Fe_3_O_4_ for CO oxidation for 14 hours. Held at 331 ⁰C after oxidative pre-treatment in O_2_ for 1 hour at 450 ⁰C.


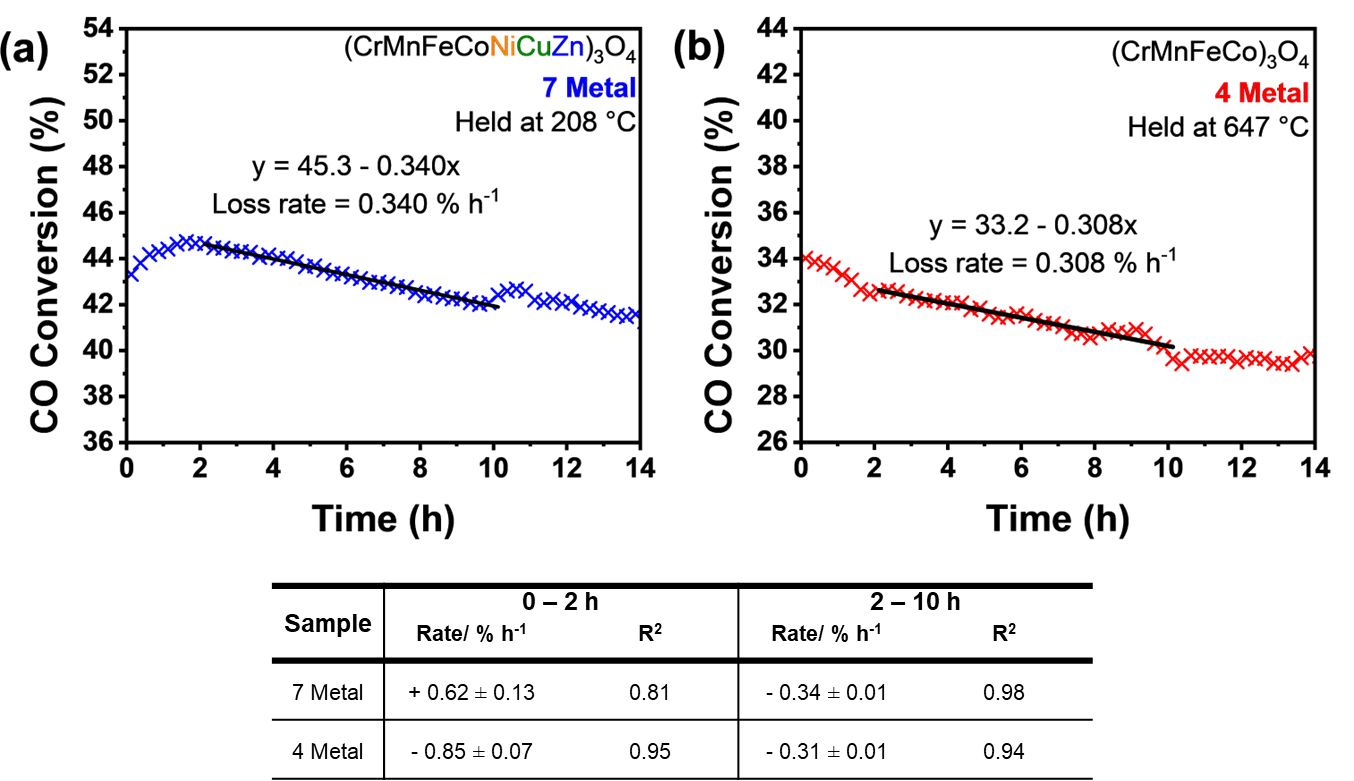


**Figure S26.** Linear fitting of (a) 7-metal and (b) 4-metal stability studies between 2 h and 10 h to obtain CO conversion % loss rates with extracted line equations and R^2^ values shown. The table summarizes this information and the change in the percentage CO conversion per h between 0 and 2 h. To allow a sufficient fit, raw data has been averaged over 15 min windows.

Table S6. Summary of synthetic procedures, catalyst pre-treatment, feeds, T_10_, T_50_ values, and stability assessments for this work compared to similar literature of HEO or monometallic spinels over CO oxidation.^[4]^

| **Ref** | **Sample** | **Synthesis** | **Catalyst**  **Pre-treatment** | **Catalyst**  **Feed** | **T_10_ /°C** | **T_50_ /°C** | **Stability** |
| --- | --- | --- | --- | --- | --- | --- | --- |
| Xie *et al.* 2009 ^[4a]^ | Co_3_O_4_ nanorods | Solution processed and annealed in air for 4 h at 450 °C | 20 vol. % O_2_/Ar at 450 °C for 1 h | 1 vol. % CO, 2.5 vol. % & He balance.  Rate: 50 mL min^-1^ | - | < -77 | - |
| Oliveira *et al.* 2004 ^[4b]^ | Fe_3_O_4_ | Precipitation from nitrate and NH_4_OH solutions. Dried at 100 °C for 12 h and annealed in N_2_ at 400 °C for 2 h | - | 25 vol. % CO & dry air balance  Rate: 50 mL min^-1^ | ~60 | ~370 | Fe_3_O_4_ oxidised at high temperature to γ-Fe_2_O_3_ |
| Hudy *et al.* 2022 ^[4c]^ | (Cr_0.22_Co_0.78_)[Co_1.15_Cr_0.85_]O_4_ | Sol gel with metal nitrates.  Pre-heated at 400 °C for 15 mins, ground then heated at 600 °C for 4 h | 5 vol. % O_2_ at 600 °C | 2 vol. % CO in He  Rate: 100 mL min^-1^ | ~180 | ~220 | - |
|  | (Mn_0.11_Co_0.89_)[Co_1.03_Mn_0.97_]O_4_ |  |  |  | ~210 | ~225 |  |
|  | Co_3_O_4_ |  |  |  | ~145 | ~185 |  |
| Xu *et al.* 2020 ^[4d]^ | CeZrHfTiLaO_x_ | Ball milled for 1 h. Post annealed 900 °C for 4 h.  Ramp rate: 2 °C min^-1^ | - | 1 vol.% CO & air balance | ~237 | ~280 | Little change to Pd@CeZrHfTiLaO_x_  activity at 170 °C for 40 h. |
|  | Pd@CeZrHfTiLaO_x_ |  |  |  | ~70 | ~170 |  |
| Chen *et al.* 2018 ^[4e]^ | NiMgCuZnCoO*_x_* | Hand ground and ball milled for 2 h. Post annealed 900 °C for 4 h  Ramp rate: 5 °C min^-1^ | - | 1 vol.% CO & dry air balance  Rate: 10 mL min^-1^ | - | 256 | Little change to PtNiMgCuZnCoO_x_ activity at 135 °C for 100 h. |
|  | PtNiMgCuZnCoO_x_ |  |  |  | - | ~210 |  |
| Zhang *et al.* 2023 ^[4f]^ | (MnCuCo_3_NiFe)*_x_*O*_y_* | Solvothermal method using nitrate.  16 h at 180 °C, dried > 6 h & heated 500 °C at 2 h  Ramp rate: 1 °C min^-1^ | - | 1 vol.% CO, 20 vol.% O_2_ & N_2_ balance  Rate: 10 mL min^-1^ | ~80 | ~105 | HESO was found to better resist SO_2_ and H_2_O poisoning than Co_3_O_4_ |
| Zhang *et al.* 2024 ^[4g]^ | (ZnMgCuMnCo)Al_2_O_4_ | Ball milled for 1 h.  Post annealed 600 °C for 6 h.  Ramp rate: 5 °C min^-1^ | - | 1 vol.% CO & dry air balance | ~145 | 210 | HESO was found to better resist SO_2_ poisoning than unary spinels |
| **This Work** | Fe_3_O_4_ | Solventless Thermolysis of molecular precursors at 900 °C,4 h, Air  Ramp Rate: 15 °C min^-1^ | 20 vol. % O_2_/Ar at 450 °C for 1 h | 1 vol.% CO, 5 vol.% O_2_ & Ar balance  Rate: 50 mL min^-1^ | 285 | 371 | 4-metal lost more activity than the 7-metal during 14 h of operation |
|  | (CrMnFeCo)_3_O_4_ |  |  |  | 454 | - |  |
|  | (CrMnFeCoNi)_3_O_4_ |  |  |  | 392 | 569 |  |
|  | (CrMnFeCoNiCu)_3_O_4_ |  |  |  | 316 | 478 |  |
|  | (CrMnFeCoNiCuZn)_3_O_4_ |  |  |  | 168 | 250 |  |

# **References**

[1] J. Qu, A. Elgendy, R. Cai, M. A. Buckingham, A. A. Papaderakis, H. de Latour, K. Hazeldine, G. F. S. Whitehead, F. Alam, C. T. Smith, D. J. Binks, A. Walton, J. M. Skelton, R. A. W. Dryfe, S. J. Haigh, D. J. Lewis, *Adv. Sci.* **2023**, *10* (14), e2204488, <https://doi.org/10.1002/advs.202204488>.

[2] a) B. Ward-O'Brien, P. D. McNaughter, R. Cai, A. Chattopadhyay, J. M. Flitcroft, C. T. Smith, D. J. Binks, J. M. Skelton, S. J. Haigh, D. J. Lewis, *Nano Lett.* **2022**, *22* (20), 8045, <https://doi.org/10.1021/acs.nanolett.2c01596>; b) B. Ward-O'Brien, E. J. Pickering, R. Ahumada-Lazo, C. Smith, X. L. Zhong, Y. Aboura, F. Alam, D. J. Binks, T. L. Burnett, D. J. Lewis, *J. Am. Chem. Soc.* **2021**, *143* (51), 21560, <https://doi.org/10.1021/jacs.1c08995>.

[3] G. H. J. Johnstone, M. U. Gonzalez-Rivas, K. M. Taddei, R. Sutarto, G. A. Sawatzky, R. J. Green, M. Oudah, A. M. Hallas, *J. Am. Chem. Soc.* **2022**, *144* (45), 20590, <https://doi.org/10.1021/jacs.2c06768>.

[4] a) X. Xie, Y. Li, Z. Q. Liu, M. Haruta, W. Shen, *Nat.* **2009**, *458* (7239), 746, <https://doi.org/10.1038/nature07877>; b) S. Oh, G. Hoflund, *J. Catal.* **2007**, *245* (1), 35, <https://doi.org/10.1016/j.jcat.2006.09.016>; c) C. Hudy, O. Długosz, J. Gryboś, F. Zasada, A. Krasowska, J. Janas, Z. Sojka, *Catal. Sci. Technol.* **2022**, *12* (8), 2446, <https://doi.org/10.1039/d2cy00388k>; d) H. Xu, Z. Zhang, J. Liu, C. L. Do-Thanh, H. Chen, S. Xu, Q. Lin, Y. Jiao, J. Wang, Y. Wang, Y. Chen, S. Dai, *Nat. Commun.* **2020**, *11* (1), 3908, <https://doi.org/10.1038/s41467-020-17738-9>; e) H. Chen, J. Fu, P. Zhang, H. Peng, C. W. Abney, K. Jie, X. Liu, M. Chi, S. Dai, *J. Mater. Chem. A* **2018**, *6* (24), 11129, <https://doi.org/10.1039/c8ta01772g>; f) M. Zhang, X. Duan, Y. Gao, S. Zhang, X. Lu, K. Luo, J. Ye, X. Wang, Q. Niu, P. Zhang, S. Dai, *ACS Appl. Mater. Interfaces.* **2023**, *15* (39), 45774, <https://doi.org/10.1021/acsami.3c07268>; g) M. Zhang, Y. Gao, C. Xie, X. Duan, X. Lu, K. Luo, J. Ye, X. Wang, X. Gao, Q. Niu, P. Zhang, S. Dai, *Nat. Commun.* **2024**, *15* (1), 8306, <https://doi.org/10.1038/s41467-024-52531-y>.
